# Classification of missense variants in the *N*-methyl-d-aspartate receptor *GRIN* gene family as gain- or loss-of-function

**DOI:** 10.1093/hmg/ddad104

**Published:** 2023-06-27

**Authors:** Scott J Myers, Hongjie Yuan, Riley E Perszyk, Jing Zhang, Sukhan Kim, Kelsey A Nocilla, James P Allen, Jennifer M Bain, Johannes R Lemke, Dennis Lal, Timothy A Benke, Stephen F Traynelis

**Affiliations:** Department of Pharmacology and Chemical Biology, Emory University School of Medicine, Atlanta, GA 30322, USA; The Center for Functional Evaluation of Rare Variants (CFERV), Emory University School of Medicine, Atlanta, GA 30322, USA; Department of Pharmacology and Chemical Biology, Emory University School of Medicine, Atlanta, GA 30322, USA; The Center for Functional Evaluation of Rare Variants (CFERV), Emory University School of Medicine, Atlanta, GA 30322, USA; Department of Pharmacology and Chemical Biology, Emory University School of Medicine, Atlanta, GA 30322, USA; Department of Pharmacology and Chemical Biology, Emory University School of Medicine, Atlanta, GA 30322, USA; Department of Pharmacology and Chemical Biology, Emory University School of Medicine, Atlanta, GA 30322, USA; Department of Pharmacology and Chemical Biology, Emory University School of Medicine, Atlanta, GA 30322, USA; Department of Pharmacology and Chemical Biology, Emory University School of Medicine, Atlanta, GA 30322, USA; The Center for Functional Evaluation of Rare Variants (CFERV), Emory University School of Medicine, Atlanta, GA 30322, USA; Department of Neurology, Division of Child Neurology, Columbia University Irving Medical Center, New York, NY 10032, USA; Institute of Human Genetics, University of Leipzig Medical Center, Leipzig 04103, Germany; Center for Rare Diseases, University of Leipzig Medical Center, Leipzig 04103, Germany; Genomic Medicine Institute, Lerner Research Institute, Cleveland Clinic, Cleveland, OH 44106, USA; Epilepsy Center, Neurological Institute, Cleveland Clinic, Cleveland, OH 44195, USA; Stanley Center for Psychiatric Research, Broad Institute of MIT and Harvard, Cambridge, MA 02142, USA; Cologne Center for Genomics (CCG), Medical Faculty of the University of Cologne, Köln 50923, Germany; Department of Pediatrics, Pharmacology and Neurology, University of Colorado School of Medicine, and Children’s Hospital Colorado, Aurora, CO 80045, USA; Department of Pharmacology and Chemical Biology, Emory University School of Medicine, Atlanta, GA 30322, USA; The Center for Functional Evaluation of Rare Variants (CFERV), Emory University School of Medicine, Atlanta, GA 30322, USA; Emory Neurodegenerative Disease Center, Emory University School of Medicine, Atlanta, GA 30322, USA

## Abstract

Advances in sequencing technology have generated a large amount of genetic data from patients with neurological conditions. These data have provided diagnosis of many rare diseases, including a number of pathogenic *de novo* missense variants in *GRIN* genes encoding *N*-methyl-d-aspartate receptors (NMDARs). To understand the ramifications for neurons and brain circuits affected by rare patient variants, functional analysis of the variant receptor is necessary in model systems. For NMDARs, this functional analysis needs to assess multiple properties in order to understand how variants could impact receptor function in neurons. One can then use these data to determine whether the overall actions will increase or decrease NMDAR-mediated charge transfer. Here, we describe an analytical and comprehensive framework by which to categorize *GRIN* variants as either gain-of-function (GoF) or loss-of-function (LoF) and apply this approach to *GRIN2B* variants identified in patients and the general population. This framework draws on results from six different assays that assess the impact of the variant on NMDAR sensitivity to agonists and endogenous modulators, trafficking to the plasma membrane, response time course and channel open probability. We propose to integrate data from multiple *in vitro* assays to arrive at a variant classification, and suggest threshold levels that guide confidence. The data supporting GoF and LoF determination are essential to assessing pathogenicity and patient stratification for clinical trials as personalized pharmacological and genetic agents that can enhance or reduce receptor function are advanced. This approach to functional variant classification can generalize to other disorders associated with missense variants.

## Introduction

A large number of missense variants have been identified in patients with various neurological conditions, and many of these genes have been identified as monogenic risk factors that can cause or contribute to neurological disease. Among genes unambiguously associated with neurological disease, ion channels are disproportionately represented (1–5). Four genes (*GRIN1*, *GRIN2A*, *GRIN2B* and *GRIN2D*) that encode *N*-methyl-d-aspartate receptor (NMDAR) subunits (6) have been associated with *GRIN*-related disorders that belong to the top 10 diagnoses among individuals with developmental and epileptic encephalopathy (7). In addition, *GRIN2A* variants have been linked to schizophrenia (8). Hundreds of variants in these genes have been identified, and approximately half of them have some degree of functional data associated with them (6,9–13). The NMDAR is complex, and activation and subsequent current responses require the binding of two agonists (glutamate and glycine or d-serine) and relief of voltage-dependent channel block by extracellular Mg^2+^. The strength of the charge transfer that the receptor catalyzes also depends on the number of channels that reside on the plasma membrane, the time course with which the receptor is active before agonist unbinds or the receptor desensitizes, and the overall probability that the ion conducting pore of an agonist-bound receptor will be open. Thus, an assessment of the net effect of a missense variant on receptor function requires assays for at least six parameters plus a method for interpreting the magnitude of the changes observed. For example, a modest decrease in glutamate potency might suggest reduced function of the variant receptor; however, if the variant also has diminished block by extracellular Mg^2+^, this could outweigh the effects on agonist binding. Thus, a comprehensive analysis is needed to support functional conclusions.

In genes where variants classified as pathogenic by accepted American College of Human Genetics and Genomics (ACMG) criteria (14) can cause clinically similar disorders through different disease mechanisms, genetic counselors, clinicians and basic scientists evaluating potential precision therapeutic treatments rely on categorization of the effects of missense variants on protein function. On the most basic level, this can be achieved by determining whether a variant change in the protein increases or decreases its function. The long-standing terminology for this classification is to refer to variants that enhance overall actions of a protein as ‘gain-of-function’ (GoF) and variants that decrement overall action as ‘loss-of-function’ (LoF). While obviously an over-simplification of the multiple functions almost all proteins carry out, this initial characterization allows several important determinations to be made and later correlated with neurobehavioral phenotypes. Variant proteins that clearly increase or decrease function, as opposed to those with no detectable effect, are more likely to be pathogenic according to international classification guidelines (14), and this is essential for clinical diagnosis and decision making. In addition, as precision pharmacological and genetic strategies are evaluated for potential treatment of patients with a disease arising from missense variants, it is critical to know whether overall target protein function is enhanced or decreased by the variant and by what magnitude. One would not want to treat a patient with decreased protein function with a drug that further diminishes the function of the affected protein. Rather, one might seek therapeutic strategies to boost protein function or expression when missense variants decrement that function. Likewise, one would not want to enhance the function of a protein when a variant already has enhanced function of that protein. In preliminary studies, inappropriate treatment of an individual with a GoF NMDAR disorder with an agent designed to enhance function for LoF patients resulted in behavioral deterioration (15). Rather, drugs that reduce function or expression would be more likely to provide some improvement in the patient’s clinical symptoms. Further, stratifying patient populations via specific GoF or LoF inclusion and exclusion criteria for rare genetic diseases is paramount for safety and subsequent success in clinical trials because small patient population sizes necessitate smaller clinical trials that are highly sensitive to patient variability, clinical trial design and clinical outcome measures beyond epilepsy endpoints (16). That is, a clinical trial of an inhibitor on a mixed pool of study subjects with both GoF and LoF variants could yield highly variable data, decreasing the likelihood that clear determination of safety and benefit could be achieved. These categorizations as GoF or LoF provides a starting point for genetic counselors and clinicians to explain to caregivers, families and patients the nature of the diagnosis, which is critical so that they can understand the features of the variant-related conditions as well as the risks and benefits of potential treatments, whether via a clinical trial or following regulatory approvals. Lastly, understanding a more complete profile of variant receptor function via multiple assays can provide a path to eventually categorize variants based on mechanism of receptor dysfunction, which may inform future treatment options. The wide range of potential functional changes in NMDARs harboring missense variants is likely related to the highly heterogeneous clinical phenotypes. However, high-resolution phenotypic proxies for molecular dysfunction have not been well developed for NMDAR disorders, necessitating molecular/functional testing (13). This is in contrast to variants in, for example, the *SCN2A* voltage-gated channel sodium gene, for which the majority of variants produce seizure disorders, with the neonatal seizure onset being clearly correlated with GoF whereas seizures after year 2 are correlated with LoF (17). Therefore, precise GoF and LoF categorization of ion channel variants has prognostic as well as precision therapeutic implications.

While useful, the categorization as GoF and LoF has limitations. NMDARs that have altered glutamate potency will probably have a different effect on a circuit compared with those with altered Mg^2+^ sensitivity, an altered response time course or a combination of changes in multiple receptor properties. Thus, a simple binary determination of whether a variant increases or decreases NMDAR-mediated charge transfer will not speak to subtle changes in circuit function that could be important for the manifestation of the clinical phenotype. Furthermore, the determination of receptor properties may not bear on neurological or clinical symptoms that are secondary to compensatory up- or downregulation of other genetic and developmental programs that alter circuit function. However, these more complicated situations only become analytically tractable as larger numbers of patient variants for which all properties were assessed are followed in longitudinal studies, which would allow variants that change different functions to be correlated with different clinical features. This complexity, while a future opportunity to further stratify study subjects and patients (e.g. (18)), does not invalidate the utility of GoF and LoF designations as a criterion for selection of pharmacological assessments. Here, we provide new data on *GRIN2B* missense variants, propose a framework to make these GoF and LoF designations following functional analysis of multiple parameters of NMDARs harboring a missense variant, and describe an approach to determine GoF and LoF status for both new variants as well as variants described in the literature.

## Results

### Comprehensive analysis of variant properties *in vitro*

We have previously analyzed the functional properties of NMDAR missense variants using voltage clamp assays of recombinant receptors harboring a mutation in cDNA that matches the missense variant (GRIN Portal, CFERV). These assays have utilized two heterologous expression systems (*Xenopus laevis* oocytes and transfected mammalian fibroblasts) that allow study of a homogeneous population of receptors, are scalable in terms of accessibility and expense, and have been shown to yield receptor properties that match those found in neurons (6,19). Oocytes allow rapid determination of the potency of glutamate, glycine, Mg^2+^, Zn^2+^ and NMDAR inhibitors and modulators as well as estimates of receptor open probability. However, oocyte recordings have low temporal resolution given their large size and vitelline membrane, which slows solution exchange. Therefore, we also conduct patch clamp recordings in response to submillisecond application of agonists in transfected mammalian cells to define the response time course. We additionally use transfected mammalian cells to biochemically estimate variant effects on receptor trafficking to the plasma membrane. Although expression of variants in native systems offers some advantages, expression in cultured neurons would create variable and mixed populations of variant and wild-type (WT) NMDARs, obscuring variant properties. In addition, neurons are not amenable to rapid solution exchange, at least an order of magnitude more resource and time intensive to prepare, and difficult to transfect, complicating complete characterization of large numbers of NMDAR variants.

**Figure 1:**
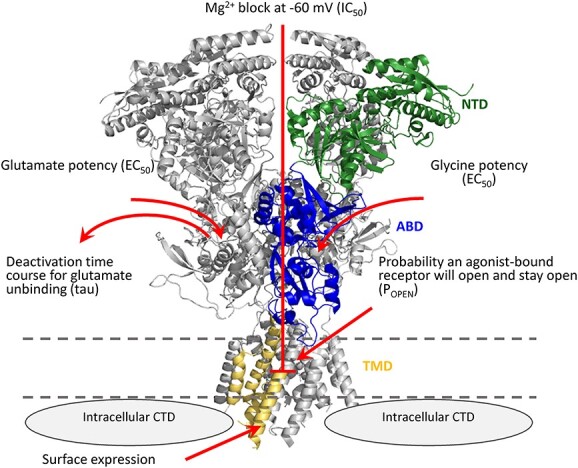
Assays used to determine GoF and LoF characteristics of missense *GRIN* variants and assess charge transfer. A structure of the GluN1/GluN2B NMDAR is shown from Karakas and Furukawa (65) with one subunit colored to illustrate the amino terminal domain (NTD, green; also known as ATD), agonist binding domain (ABD, blue; also known as ligand binding domain, LBD), the transmembrane domain (TMD, gold) and intracellular C-terminal domain (CTD, gray). The red arrows show the six assays performed.

**Figure 2:**
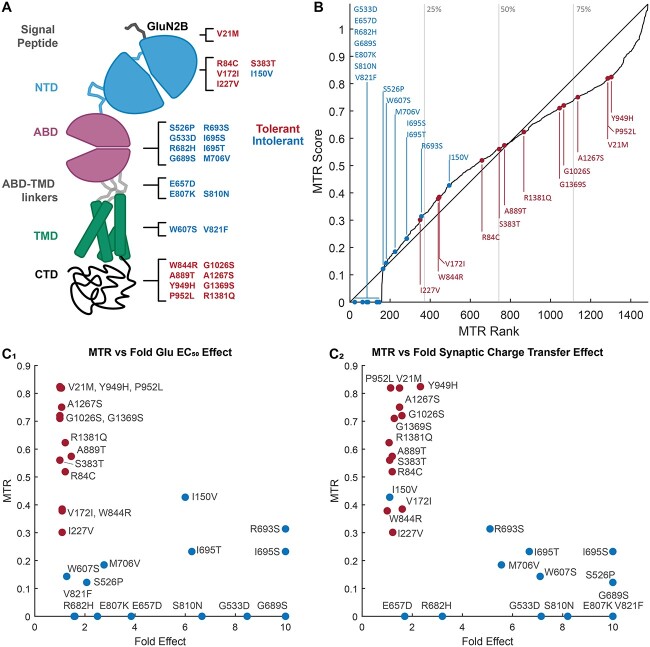
Location of patient-derived and benign gnomAD-derived variants. (**A**) The domains of an NMDAR subunit are shown with new variants from patients (blue) and gnomAD (red) in this study listed. (**B**) Rank order of MTR intolerance score (the 3DMTR, closest 31 residues intra-subunit, is used where possible, otherwise the 1DMTR score, 31 residues smoothing window, is used) for all residues in all subunits the NMDAR complex, including the intracellular C-terminal domain. The location of each variant reported in this study within this ranking is shown. Gray lines indicate quartiles. (**C**) A plot of the MTR score for a given variant (red gnomAD, blue patient-derived) and the fold shift in glutamate EC_50_ (C_1_) or synaptic charge transfer (C_2_) is shown.

**Figure 3:**
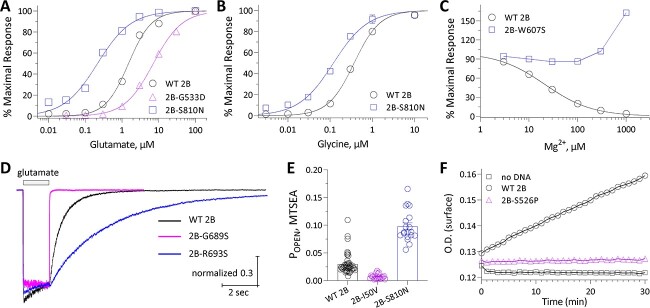
Effects of *GRIN* variants on functional properties of NMDARs. The effect of the indicated representative *GRIN2B* variants are shown for glutamate potency (**A**), glycine potency (**B**), Mg^2+^ potency (**C**), deactivation time course following rapid removal of glutamate (**D**), channel open probability (**E**) and receptor surface expression (**F**).

Here, we have implemented assays of recombinant receptors to determine the EC_50_ for the agonists glutamate and glycine, IC_50_ for inhibition of the NMDAR by extracellular Mg^2+^, open probability for an agonist-bound receptor, response time course following the rapid removal of the agonist glutamate on a time scale relevant for synaptic transmission, and surface expression (20–31) (Fig. 1; see Materials and Methods). We have functionally assessed 14 previously unstudied *GRIN2B* missense variants identified in patients, reported in ClinVar, and absent from gnomAD (V2.1.1 Non-neuro, accessed 10 April 2023). We used 3DMTR (32) to assess the intolerance of regions in the receptor in which these variants are located when structural data existed, otherwise 1DMTR was used (Fig. 2). Analysis of patient-derived variants yielded a wide range of results, with single or multiple parameters changing in a manner that should increase or decrease overall synaptic function. Figure 3 shows the results from representative variants illustrating different effects on NMDAR functional properties. Table 1 summarizes the outcome of these assays for each variant. For comparison, we also applied these same assays to 13 benign *GRIN2B* variants located in more tolerant domains in healthy individuals (Fig. 2). As expected, NMDARs harboring benign variants showed properties similar to WT receptors (Table 2; Fig. 2C).

**Table 1:** Functional properties of *GRIN2B* patient-derived variants

*GRIN2B* variant	Glu EC_50_, μm, 95% CI, *n*	Gly EC_50_, μm, 95% CI, *n*	Mg^2+^ IC_50_, μm, 95% CI, *n*	*P*_OPEN_, MTSEA, mean ± SEM (*n*)	*τ*_W_, ms, mean ± SEM (n)	Surface/total, mean ± SEM (n)
WT	1.2 [1.0, 1.4], 24	0.28 [0.24, 0.32], 12	31 [19, 52], 11	0.022 ± 0.002 (23)	706 ± 35 (9)	1.0 (3)
I150V	0.20 [0.18, 0.24], 18^*^	0.19 [0.13, 0.26], 12	22 [18, 27], 12	0.007 ± 0.001 (21)^*^	3460 ± 732 (4)^*^	1.4 ± 0.20 (4)
WT	1.4 [1.3, 1.6], 14	0.31 [0.26, 0.38], 10	13 [10, 18], 10	0.028 ± 0.002 (9)	706 ± 35 (9)	1.0 (4)
S526P	2.9 [2.6, 3.2], 10^*^	0.28 [0.23, 0.35], 14	11 [9.0, 14], 11	0.028 ± 0.0006 (19)	<350 *^a^*	0.10 ± 0.04 (5)^*^
WT	0.98 [0.79, 1.2], 12	0.28 [0.24, 0.32], 12	31 [19, 52], 11	0.028 ± 0.0025 (9)	706 ± 35 (9)	1.0 (2)
G533D	8.3 [6.2, 11], 11^*^	0.49 [0.41, 0.59], 13^*^	29 [21, 41], 10	0.016 ± 0.0014 (19)^*^	277 ± 10 (4)^*^	0.72 ± 0.27 (3)
WT	1.1 [0.96, 1.3], 12	0.36 [0.32, 0.43], 12	20 [14, 23], 11	0.034 ± 0.0027 (34)	706 ± 35 (9)	1.0 (4)
W607S	1.4 [1.2, 1.7], 11	0.56 [0.49, 0.65], 11^*^	>1000, 11^*^	0.015 ± 0.0034 (14)^*^	508 ± 69 (5)	0.62 ± 0.17 (3)^*^
WT	1.0 [0.85, 1.26], 12	0.28 [0.24, 0.33], 12	26 [22, 32], 10	0.022 ± 0.002 (23)	706 ± 35 (9)	1.0 (6)
E657D	0.26 [0.21, 0.32], 10^*^	0.14 [0.11, 0.17], 10^*^	26 [23, 29], 10	0.0087 ± 0.0007 (14)^*^	2480 ± 196 (7)^*^	1.01 ± 0.25 (5)
WT	0.96 [0.85, 1.1], 14	0.24 [0.21, 0.28], 11	18 [15, 20] 11	0.041 ± 0.0054 (19)	706 ± 35 (9)	1.0 (6)
R682H	0.60 [0.54, 0.67], 12^*^	0.15 [0.12, 0.18], 11^*^	27 [21, 35], 11^*^	0.049 ± 0.0067 (30)	1040 ± 121 (4)^*^	1.26 ± 0.68 (3)
WT	1.0 [0.73, 1.4], 10	0.28 [0.23, 0.35], 12	32 [24, 43], 7	0.035 ± 0.0025 (10)	706 ± 35 (9)	1.0 (4)
G689S	>5810, 12^*^	0.44 [0.39, 0.51], 12^*^	21 [18, 26], 10	0.049 ± 0.014 (7)	7.8 ± 0.48 (6)^*^	0.78 ± 0.03 (4)^*^
WT	1.4 [1.3, 1.6], 19	0.36 [0.31, 0.43], 14	25 [15, 39], 7	0.033 ± 0.0032 (15)	706 ± 35 (9)	1.0 (4)
R693S	0.10 [0.05, 0.19], 13^*^	0.32 [0.23, 0.38], 12	17 [15, 20], 12	0.050 ± 0.0057 (25)	5530 ± 605 (5)^*^	0.70 ± 0.03 (4)^*^
WT	0.96 [0.84, 1.1], 18	0.43 [0.37, 0.50], 6	26 [21, 31], 6	0.035 ± 0.0025 (10)	706 ± 35 (9)	1.0 (6)
I695S	105 [52, 213], 15^*^	0.33 [0.31, 0.35], 6^*^	24 [21, 27], 6	0.017 ± 0.0039 (10)^*^	252 ± 33 (9)^*^	0.34 ± 0.07 (4)^*^
WT	0.99 [0.88, 1.1], 12	0.37 [0.31, 0.44], 13	38 [26, 56], 10	0.035 ± 0.0025 (10)	706 ± 35 (9)	1.0 (4)
I695T	6.2 [5.0, 7.6], 12^*^	0.32 [0.28, 0.37], 12	27 [20, 42], 11	0.014 ± 0.0011 (18)^*^	351 ± 27 (4)^*^	0.67 ± 0.05 (4)^*^
WT	1.05 [0.96, 1.1], 21	0.30 [0.26, 0.35], 14	13 [9, 19], 25	0.024 ± 0.0017 (10)	706 ± 35 (9)	1.0 (6)
M706V	2.9 [2.2, 3.8], 19^*^	0.26 [0.23, 0.30], 13	23 [14, 36], 25	0.016 ± 0.00065 (12)^*^	484 ± 22 (5)^*^	0.32 ± 0.06 (3)^*^
WT	1.4 [1.1, 1.9], 12	0.42 [0.32, 0.54], 12	36 [20, 66], 6	0.035 ± 0.0025 (10)	706 ± 35 (9)	1.0 (6)
E807K	3.5 [2.9, 4.0], 12^*^	0.55 [0.46, 0.67], 12	17 [15, 20], 12	0.013 ± 0.0025 (20)^*^	199 ± 25 (6)^*^	0.87 ± 0.10 (6)
WT	1.4 [1.3, 1.6], 19	0.37 [0.33, 0.42], 18	25 [15, 40], 7	0.028 ± 0.0025 (9)	706 ± 35 (9)	1.0 (4)
S810N	0.21 [0.16, 0.26], 14^*^	0.12 [0.10, 0.15], 14^*^	25 [18, 34], 10	0.098 ± 0.0065 (19)^*^	2620 ± 373 (6)^*^	0.78 ± 0.07 (4)^*^
WT	1.24 [1.1, 1.4], 20	0.35 [0.33, 0.37], 10	19 [15, 23], 16	0.039 ± 0.0032 (18)	706 ± 35 (9)	1.0 (4)
V821F	0.79 [0.60, 1.0], 14^*^	0.17 [0.13, 0.23], 14^*^	15 [13, 18], 12	0.011 ± 0.0021 (17)^*^	84 ± 16 (4)^*^	1.19 ± 0.13 (4)

The l-glutamate (Glu) concentration–response relationship was determined in the presence of maximally effective glycine (e.g. 100 μm), and the glycine (Gly) concentration–response relationship was determined in the presence of maximally effective glutamate (e.g. 100 μm). Wild-type IC_50_ and EC_50_ values were from experiments performed the same day. Data shown are the mean IC_50_ or EC_50_ value with 95% confidence intervals determined from the LogEC_50_ or LogIC_50_ values; ^*^indicates 95% confidence intervals that are non-overlapping with WT GluN2B-containing NMDARs, which corresponds to *P* < 0.01. Data are mean ± SEM for *P*_OPEN_, deactivation weighted tau (*τ*_w_) and beta-lac surface/total ratio; *n* is the number of cells recorded from. Weighted tau deactivation (*τ*_w_) was determined from the brief application of l-glutamate. *^a^*Only 1 of 36 HEK cells responded, consistent with low expression in surface assay. Each pair of rows (e.g. the first WT and I150V) are results from same day recordings.

**Table 2:** *GRIN2B* gnomAD-derived benign variants

***GRIN2B* variant**	**Allelic frequency**	**Glu EC**_**50**_ **, μm, 95% CI, *n***	**Gly EC**_**50**_ **, μm, 95% CI, *n***	**Mg^2+^ IC**_**50**_ **, μm, 95% CI, *n***	***P***_**OPEN**_ **, MTSEA, mean ± SEM, (*n*)**	***τ***_**W**_ **, ms, mean ± SEM (*n*)**	**Surface/total, mean ± SEM (*n*)**
WT	—	1.6 [1.4, 1.9], 12	0.52 [0.40, 0.67], 12	26 [21, 32], 11	0.031 ± 0.0014 (12)	779 ± 32 (11)	1.0 (4)
V21M	54/247738	1.7 [1.4, 2.0], 12	0.59 [0.52, 0.67], 12	18 [13, 25], 10	0.033 ± 0.0014 (19)	683 ± 71 (4)	0.98 ± 0.04 (4)
WT	—	1.4 [1.2, 1.6], 12	0.31 [0.29, 0.34], 12	26 [19, 35], 11	0.028 ± 0.0025 (9)	779 ± 32 (11)	1.0 (8)
R84C	2/282442	1.7 [1.4, 2.1], 12	0.28 [0.25, 0.33], 12	23 [20, 26], 12	0.038 ± 0.0018 (18)^*^	785 ± 23 (6)	0.61 ± 0.14 (4)^*^
WT	—	1.1 [1.0, 1.2], 12	0.33 [0.26, 0.41], 11	17 [13, 23], 12	0.031 ± 0.0014 (12)	779 ± 32 (11)	1.0 (4)
V172I	8/282254	1.2 [1.0, 1.4], 12	0.30 [0.26, 0.35], 12	17 [14, 21], 12	0.040 ± 0.0044 (14)	557 ± 25 (4)	0.95 ± 0.10 (4)
WT	—	1.2 [0.97, 1.4], 11	0.32 [0.27, 0.38], 10	21 [15, 29], 10	0.031 ± 0.0014 (12)	779 ± 32 (11)	1.0 (6)
I227V	7/251216	1.1 [0.90, 1.4], 13	0.23 [0.18, 0.30], 11	18 [15, 20], 12	0.030 ± 0.0010 (13)	760 ± 106 (4)	1.3 ± 0.38 (8)
WT	—	1.2 [1.1, 1.4], 16	0.49 [0.34, 0.72], 12	21 [16, 27], 11	0.044 ± 0.0044 (10)	779 ± 32 (11)	1.0 (6)
S383T	15/282804	1.2 [0.95, 1.6], 13	0.28 [0.24, 0.32], 11^*^	25 [17, 35], 12	0.036 ± 0.0014 (20)	808 ± 63 (4)	1.1 ± 0.13 (6)
WT	—	1.1 [0.88, 1.3], 12	0.37 [0.32, 0.42], 12	22 [18, 27], 12	0.044 ± 0.0044 (10)	779 ± 32 (11)	1.0 (4)
W844R	5/251492	1.2 [1.0, 1.4], 12	0.41 [0.36, 0.47], 12	21 [18, 24], 12	0.048 ± 0.0035 (17)	699 ± 67 (4)	0.84 ± 0.05 (4)^*^
WT	—	1.6 [1.1, 2.2], 11	0.44 [0.35, 0.53], 10	25 [21, 30], 14	0.044 ± 0.0044 (10)	779 ± 32 (11)	1.0 (5)
A889T	5/282508	1.1 [0.78, 1.5], 10	0.42 [0.37, 0.48], 10	21 [16, 29], 10	0.045 ± 0.0029 (15)	834 ± 41 (5)	1.0 ± 0.18 (4)
WT	—	1.6 [1.4, 1.9], 12	0.52 [0.40, 0.68], 12	26 [21, 32], 11	0.044 ± 0.0044 (10)	779 ± 32 (11)	1.0 (8)
Y949H	2/251496	1.6 [1.5, 1.7], 12	0.54 [0.47, 0.62], 12	15 [11, 20], 12^*^	0.040 ± 0.0024 (17)	807 ± 89 (4)	0.78 ± 0.06 (4)^*^
WT	—	0.64 [0.50, 0.80], 11	0.22 [0.18, 0.27], 12	47 [32, 69], 12	0.044 ± 0.0044 (10)	779 ± 32 (11)	1.0 (4)
P952L	2/251492	0.65 [0.51, 0.84], 12	0.25 [0.21, 0.31], 12	51 [39, 67], 12	0.058 ± 0.0077 (16)	642 ± 28 (4)^*^	0.73 ± 0.02 (4)^*^
WT	—	1.2 [1.1, 1.4], 14	0.32 [0.30, 0.36], 12	20 [16, 27], 11	0.033 ± 0.0031 (7)	779 ± 32 (11)	1.0 (4)
G1026S	61/282800	1.2 [1.1, 1.4], 12	0.30 [0.26, 0.33], 12	25 [15, 39], 10	0.033 ± 0.0010 (17)	680 ± 101 (4)	0.67 ± 0.18 (4)
WT	—	0.91 [0.81, 1.0], 10	0.32 [0.28, 0.38], 12	26 [20, 33], 12	0.033 ± 0.0031 (7)	779 ± 32 (11)	1.0 (4)
A1267S	68/282766	0.85 [0.75, 0.96], 12	0.32 [0.29, 0.35], 12	23 [16, 34], 12	0.031 ± 0.0009 (14)	883 ± 51 (3)	0.62 ± 0.22 (3)
WT	—	1.2 [1.0, 1.4], 10	0.32 [0.27, 0.38], 10	21 [15, 29], 10	0.033 ± 0.0031 (7)	779 ± 32 (11)	1.0 (10)
G1369S	24/251442	1.2 [1.1, 1.4], 13	0.31 [0.26, 0.36], 12	24 [18, 32], 9	0.034 ± 0.0017 (16)	715 ± 96 (4)	0.95 ± 0.17 (10)
WT	—	1.4 [1.2, 1.5], 11	0.39 [0.35, 0.45], 12	30 [23, 39], 11	0.033 ± 0.0031 (7)	779 ± 32 (11)	1.0 (4)
R1381Q	2/251454	1.7 [1.5, 1.9], 12	0.41 [0.38, 0.44], 12	41 [24, 70], 14	0.032 ± 0.0010 (10)	633 ± 25 (4)	0.91 ± 0.06 (4)

The l-glutamate (Glu) concentration–response relationship was determined in the presence of maximally effective glycine (e.g. 100 μm), and the glycine (Gly) concentration–response relationship was determined in the presence of maximally effective glutamate (e.g. 100 μm). Wild-type IC_50_ and EC_50_ values were from experiments performed the same day. Data shown are the mean IC_50_ or EC_50_ value with 95% confidence intervals determined from the logEC_50_ or logIC_50_ values; ^*^indicates 95% confidence intervals that are non-overlapping with WT GluN2B-containing NMDARs, which corresponds to *P* < 0.01. Data are mean ± SEM for *P*_OPEN_, deactivation weighted tau (*τ*_w_) and beta-lac surface/total ratio; *n* is the number of cells recorded from. Weighted tau deactivation (*τ*_w_) was determined from the brief application of l-glutamate. Each pair of rows (e.g. the first WT and V21M) are results from same day recordings.

### Choice of assays to assess GoF and LoF

We have selected six properties as necessary to assess overall receptor function: EC_50_ for glutamate and glycine, IC_50_ for Mg^2+^, open probability, response time course and surface expression. Because a change in any one of the six parameters we assessed can influence the current NMDARs mediate in neurons, we would argue that analysis of all six parameters by some means is necessary before classification of a variant as GoF or LoF, although exceptions exist (see in the following text). There are additional parameters that could influence NMDAR function that we have not included. For example, NMDAR desensitization could occur during high-frequency synaptic transmission or during accumulation of extracellular glutamate in the interstitial space during either normal synchronous firing associated with learning or pathological hypersynchronous epileptiform activity. Thus, variants that alter the rate of desensitization could impact the NMDAR contribution to circuit function (33). However, we have omitted this because it is unclear how often shallow and relatively slow NMDAR desensitization impacts normal monosynaptic transmission. In addition, the intracellular C-terminal domain controls NMDAR trafficking and postsynaptic localization, and it is a site for post-translational modifications and binding of signaling molecules such as CaMKII or calmodulin (6,19,34–36). These interactions are specific to a neuronal context that is not in heterologous expression systems currently used in these analyses. We have omitted analysis of C-terminal functions in our evaluation of variant effects for three reasons. First, the C-terminal domain is generally tolerant to variation in the healthy population, suggesting that it is not a site at which variants commonly trigger neurological disease (21,26,32,37). Second, consistent with tolerance, the C-terminal domain appears to be spared of *de novo* missense variants in patient populations (21,27,37). However, there are regions of intolerance, suggesting that some potential pathogenic C-terminal domain variants can modify receptor function or localization (38,39). Third, existing assays to assess C-terminal domain-driven biochemical changes are not scalable to hundreds of variants that are known. It will be necessary to design higher throughput assays that can be applied across many variants in a neuronal context to assess how variation in the C-terminal domain can impact receptor subcellular location and function. Finally, GluN2A-containing NMDARs harbor a high affinity Zn^2+^ binding site in the distal amino terminal domain that can inhibit the receptor response (6), which has been shown to have important physiological consequences (40). Whereas variants within the amino terminal domain can influence Zn^2+^ binding to its site (41), we have omitted this assay from variant classification determination given the general tolerance to variation of the amino terminal domain and the absence of strong Zn^2+^ inhibition on receptors lacking GluN2A. However, we advocate collecting data on Zn^2+^ sensitivity (26,31,42,43) for any variant in GluN1 or GluN2A for potential future patient stratification.

It is also worth commenting that while our voltage clamp assays provide highly reproducible results, there are other assays that could assess the six parameters we discuss. For example, current amplitude in a mammalian cell reports a combination of surface expression and open probability, and in some cases acts as a surrogate to assess these two parameters together. We have avoided this approach because response amplitude distributions are typically skewed and require high numbers of observations for statistical analysis given inherent cell-to-cell variability in cDNA uptake during transient transfections. Furthermore, overexpression of NMDARs can compromise cell health or adhesion, potentially creating a situation where cells with large current amplitudes die or become detached, rendering the NMDARs in the remaining subpopulation of cells studied by electrophysiology no longer representative of variant actions. Determination of the time course for deactivation requires rapid application and removal of the agonist glutamate to mimic what postsynaptic receptors will see in terms of neurotransmitter profile in the synaptic cleft. However, this is a specialized technique that requires a rapid perfusion system and is not necessarily accessible to all laboratories. Multiple reports support the general idea that there is a correlation between glutamate EC_50_ and the deactivation time course (44–46). Thus, one could estimate the relative change in deactivation time course from the magnitude of the change in glutamate EC_50_, with appreciation of the important caveat that this is not a direct measurement and a small number of variants appear to uncouple these two parameters. Supplementary Material, Fig. S1 shows this relationship between EC_50_ and deactivation for published *GRIN1*, *GRIN2A*, *GRIN2B* and *GRIN2D* variants, and demonstrates how one might approximate relative changes in weighted tau deactivation (*τ*_w_) from measured changes in the glutamate EC_50_. Importantly, glutamate unbinding is not the only factor determining the deactivation time course, so this approach will miss variants that change the deactivation time course by other means. In terms of surface expression, there are a myriad of techniques that could be employed including (but not limited to) fusion proteins with extracellular tags or enzymes and western blots.

NMDAR complexes are tetrameric assemblies of two GluN1 and two GluN2 subunits (2A, 2B, 2C or 2D) (6). In patients that are heterozygous for a *GRIN* variant, only one of these subunits in a receptor complex likely will be affected. The assays described here for diheteromeric receptors are predominantly performed with two copies of the variant-containing subunit. Thus, we expect to always see larger effects in expression systems than in neurons that have one variant copy within an NMDAR. Studies *in vivo* using knock-in animal models bear this prediction out, and synaptic properties, while qualitatively similar to what was observed *in vitro*, often show more modest effects (e.g. (2,47,48)). While assays with only one copy of a variant subunit in the tetrameric NMDARs are feasible and have been reported (21,23,26,42,43,49), these assays increase complexity, time and cost and thus at this time are not scalable for all known variants.

### Discrete assessment of functional parameters as a means to call GoF or LoF

Determination of pathogenicity for missense variants typically utilizes a composite score across a number of parameters based on the ACMG criteria (14). Originally, the variant classification criteria were assigned using categorial or binary variables; however, more recently, several projects have proposed that quantitative assignment of criteria using Bayesian statistical evaluation improves variant classification (50–52). The overall premise is that there are variant-driven meaningful differences in overall receptor function that are associated with relevant changes in circuit function, which influence clinically important characteristics in affected individuals. Using this strategy, we sought to determine first whether a missense variant produced a meaningful change in any aspect of function, which would increase the likelihood that the variant is pathogenic. We then considered how scored parameters could be assessed in a composite fashion to determine their potential to drive GoF versus LoF. The starting point for discrete assessment of parameters was identification of a threshold for each of the six functional parameters (glutamate EC_50_, glycine EC_50_, Mg^2+^ IC_50_, *τ*_w_ deactivation, open probability and surface expression) that provides a ‘High’ or a ‘Moderate’ level of confidence that a variant has altered that aspect of receptor function in a meaningful capacity. We selected thresholds as multiples of the standard deviation (SD) obtained from same day assays on WT NMDARs for published data (drawn from published variants in Supplementary Material, Table S1) that would provide different degrees of confidence that a value outside this range was unlikely to arise by chance. Table 3 summarizes mean values and the variability of our same-day WT NMDAR assays for these parameters across multiple independent experiments, and shows the fold change for different multiples of the SD. We selected thresholds for these assays with high confidence (99.7% of data will fall within 3 SD of the mean) or moderate confidence (95% of data falls within 2 SD of the mean). Table 4 shows the thresholds determined by the variability of same-day control experiments. By calculating the fold change for each variant parameter and comparing it with these WT-derived thresholds, we can identify how many of the parameters changed with high or moderate confidence, providing a means to determine whether the variant-containing receptor is indeed functionally altered, and a means to assess possible or likely GoF or LoF.

**Table 3:** Variability of assays used to set threshold confidence levels

				**Fold change**			
**Assay**	**Receptor**	**Mean**	**Variability**	**LoF**	**LoF**	**GoF**	**GoF**
				**3 × SD**	**2 × SD**	**2 × SD**	**3 × SD**
Glutamate EC_50_*^a^*	GluN1/GluN2A	3.8 μm	log(SD) 0.117	0.45*^b^*	0.58*^b^*	1.7*^b^*	2.2*^b^*
Glycine EC_50_*^a^*	GluN1/GluN2A	1.1 μm	log(SD) 0.114	0.45*^b^*	0.59*^b^*	1.7*^b^*	2.2*^b^*
Mg^2+^ IC_50_*^a^*	GluN1/GluN2A	22 μm	log(SD) 0.113	0.46	0.59	1.7	2.2
*P*_OPEN_, MTSEA	GluN1/GluN2A	0.23	SD 0.058	0.24	0.50	1.5	1.7
Deactivation *τ*_w_	GluN1/GluN2A	56 ms	SD 13	0.30	0.53	1.5	1.7
Surface/total protein	GluN1/GluN2A	57%	SD 7.6%	0.60	0.73	1.3	1.4
Glutamate EC_50_*^a^*	GluN1/GluN2B	1.4 μm	log(SD) 0.093	0.53*^b^*	0.65*^b^*	1.5*^b^*	1.9*^b^*
Glycine EC_50_*^a^*	GluN1/GluN2B	0.37 μm	log(SD) 0.127	0.41*^b^*	0.56 *^b^*	1.8 *^b^*	2.4 *^b^*
Mg^2+^ IC_50_*^a^*	GluN1/GluN2B	20 μm	log(SD) 0.136	0.39	0.53	1.9	2.6
*P*_OPEN_, MTSEA	GluN1/GluN2B	0.031	SD 0.007	0.34	0.56	1.4	1.7
Deactivation *τ*_w_	GluN1/GluN2B	681 ms	SD 148	0.35	0.57	1.4	1.7
Surface/total protein	GluN1/GluN2B	60%	SD 7.2%	0.64	0.76	1.2	1.4

The mean value for each parameter determined for WT NMDARs in 12–39 independent experiments is shown to two significant figures. Values for WT glutamate and glycine EC_50_, Mg^2+^ IC_50_, open probability, weighted tau deactivation (*τ*_w_) were drawn from data in the published papers described in Supplementary Material, Table S1, and new data in Supplementary Material, Table S4. Values for the ratio of surface to total protein were drawn from data from published papers described in Supplementary Material, Table S1. The standard deviaton (SD) is given, which for l-glutamate, glycine and Mg^2+^ potency was determined from the log EC_50_ and log IC_50_. The fold change expected for each parameter when the mean changes by 2×SD or 3×SD was determined for either LoF or GoF.

^*^a^*^Log EC_50_ and log IC_50_ values are normally distributed.

^*^b^*^The agonist EC_50_ values have a reciprocal relationship for gain- and loss-of-function, and thus the fold change by variant was taken as the potency ratio defined as WT EC_50_/variant EC_50_. That is, an increase in variant EC_50_ value reflects a decrease in agonist potency (promoting LoF), and a decrease in variant EC_50_ value reflects an increase in agonist potency (promoting GoF).

**Table 4:** Thresholds for discrete determination of GoF and LoF

**Variant/WT**	**Support for LoF** **(high confidence)**	**Support for LoF** **(moderate confidence)**	**Support for GoF** **(high confidence)**	**Support for GoF** **(moderate confidence)**
**Glutamate potency ratio** ^***a***^	**↓** to 0.40 or more	**↓** to 0.67–0.40	**↑** to 2.5-fold or more	**↑** to 1.5–2.5-fold
**Glycine potency ratio** ^***a***^	**↓** to 0.40 or more	**↓** to 0.67–0.40	**↑** to 2.5-fold or more	**↑** to 1.5–2.5-fold
**Mg** ^**2+**^ **IC**_**50**_ **ratio**^***b***^	**↓** to 0.40 or more	**↓** to 0.67–0.40	**↑** to 2.5-fold or more	**↑** to 1.5–2.5-fold
***τ***_**w**_ **deactivation ratio**	**↓** to 0.50 or more	**↓** to 0.67–0.50	**↑** to 2-fold or more	**↑** to 1.5–2-fold
**Open probability ratio**	**↓** to 0.50 or more	**↓** to 0.67–0.50	**↑** to 2-fold or more	**↑** to 1.5–2-fold
**Surface expression ratio**	**↓** to 0.50 or more	**↓** to 0.67–0.50	**↑** to 2-fold or more	**↑** to 1.5–2-fold

The threshold changes of a parameter for the ratio between variant and WT properties to support LoF or GoF high confidence or moderate confidence are given, and were drawn from variability measures in Table 1. We chose changes of ≥2.5-fold for potency measures as high confidence, as this usually reflected a change greater than 3×SD. Changes above 1.5-fold were close to 2×SD and thus considered to be of moderate confidence. For *τ*_w_ deactivation, open probability and surface expression, we chose >2-fold changes as indicative of high confidence because these were typically beyond 3×SD, and 1.5-fold changes as moderate confidence, because these were usually greater than 2×SD.

^*^a^*^The fold change in variant to WT potency ratio was defined as WT EC_50_/variant EC_50_ because of the reciprocal relationship between potency and EC_50_. That is, an increase in variant EC_50_ value reflects a decrease in agonist potency (promoting LoF), and a decrease in variant EC_50_ value reflects an increase in agonist potency (promoting GoF).

^*^b^*^A decrease in Mg^2+^ IC_50_ value reflects an increase in potency (more Mg^2+^ inhibition, promoting LoF), and an increase in Mg^2+^ IC_50_ value reflects a decrease in potency (less Mg^2+^ inhibition, promoting GoF).

The results of some assays could possibly eliminate the need for more information. An exceptionally large reduction in function or surface expression could be taken as strong evidence of LoF. For example, if glutamate potency dropped 5000-fold, it is unlikely that receptors will be activated under normal conditions. Thus, modest 2–3-fold changes in other parameters (e.g. modest increase in open probability) would not matter if synaptic glutamate never reaches levels *in vivo* that would activate the receptor. Likewise, if current amplitudes are too small to be measured in oocytes and mammalian cells, this prevents determination of changes in other receptor properties and could be criteria for considering the variant a LoF, provided additional studies in mammalian cells confirm that receptor protein is made and present intracellularly.

The thresholds (Table 4) we propose reflect not only changes beyond confidence intervals determined from experimental variability, but also the ability of changes in properties to drive meaningful changes in receptor function. We propose that changes in agonist potency or Mg^2+^ IC_50_ greater than 2.5-fold or lower than 0.4-fold are associated with high confidence, and changes in agonist EC_50_ or Mg^2+^ IC_50_ of 1.5–2.5-fold or 0.67–0.4-fold with moderate confidence. Changes in the weighted time constant *τ*_w_ describing exponential time course of deactivation following glutamate removal or open probability (*P*_OPEN_) directly impact charge transfer. Changes in receptor function of high confidence were associated with a change in *τ*_w_ (slowing or accelerating) or *P*_OPEN_ greater than 2-fold or less than 0.5-fold, and changes in *τ*_w_ or open probability between 1.5- and 2-fold or between 0.67- and 0.5-fold were suggestive of changes of moderate confidence. Surface expression increases were rare, with decreases being more common. Thus, we also set thresholds for changes (primarily decreases) in surface expression greater than 2-fold (or <0.5-fold) as reflective of changes of high confidence, and changes between 1.5- and 2-fold (or between 0.67- and 0.5-fold) suggestive of moderate confidence.

One can use the results from these six assays to predict net changes in charge transfer quantitatively. Swanger *et al*. (21) first introduced a means to combine functional parameters to predict the overall relative effect of a variant compared with WT receptors on the synaptic charge transfer, and this was expanded in subsequent studies by including the sensitivity to extracellular Mg^2+^ (25,29,31). A derivative of this method utilizing only a subset of these assays has been described that relies on sequence alignment to use effects for one subunit to predict results for another subunit in the absence of data (53). Because the charge transfer produced by activation of NMDARs and recorded under voltage clamp is a strong predictor of excitatory drive onto neurons, it can be a predictor of net effects of a missense variant on NMDAR-mediated processes. For synaptic signaling, the charge transfer is the integral of an exponential function if we assume rapid receptor activation. For a single exponential function, this is simply the product of the response amplitude and the fitted time constant, tau (i.e. charge transfer = amplitude × tau). Thus, all that is needed to estimate the relative change between a variant and mutant is a measure of the effect of a variant relative to WT receptors of the parameters that control the synaptic response amplitude (fraction of receptors that are agonist bound, probability that an agonist-bound receptor is open, number of receptors at the cell surface and degree of Mg^2+^ block at resting membrane potential) and a measure of the relative change in time constant describing deactivation. Lester *et al*. (44) showed that the deactivation time course following rapid glutamate removal determines the synaptic time course, making the deactivation time constant an excellent predictor of synaptic response time course. Because the synaptic glutamate concentration is thought to approach 1 mm (54), often modest changes in glutamate EC_50_ do not drive appreciable change in the relative synaptic charge transfer (see Materials and Methods). However, less than 80 nm glutamate is predicted to reside outside synapses (55–58), and thus changes in EC_50_ can have profound effects on predicted extra-synaptic currents arising from steady-state glutamate levels or dynamic changes in extrasynaptic glutamate owing to spillover, which are important in normal and pathophysiological function (59). We have previously calculated non-synaptic charge transfer (see (21)), which we also determine here.

### Assignment of GoF or LoF

Using these thresholds, we considered *Likely GoF* or *Likely LoF* to be achieved if any one parameter changed with high confidence, even if no others changed with high confidence (Table 5). We also propose that when two or more parameters changed with moderate confidence in the same direction, this is reflective of *Possible LoF* or *Possible GoF*, provided there were no conflicting changes. When we could not detect any changes within these ranges, we consider the variant to have *No Detectable Effect*. If only one parameter changed with a moderate confidence or changes occur in opposing directions, the data suggest the variant has subthreshold or conflicting actions. The net relative synaptic and non-synaptic charge transfer quantitatively incorporates all changes into the net effect, and we propose to use the synaptic and non-synaptic charge transfer to re-classify conflicting and subthreshold variants as *Possible GoF* or *Possible LoF*. In order to utilize net changes in charge transfer, a threshold that provides a degree of confidence needs to be determined as supportive of GoF and LoF. We propose that a change ≥2.5-fold or ≤0.40-fold in synaptic or non-synaptic charge transfer should elevate conflicting or subthreshold variants to *Possible GoF* or *Possible LoF*, respectively, because this degree of change would exceed the unlikely possibility that small (e.g. 10–15%) changes arising from assay variability occurs in the same direction for all six parameters. Variants that did not reach threshold after discrete evaluation and the charge transfer calculations were considered to have *No Detectable Effect*, and variants with conflicting results that were not promoted to *Possible GoF* or *Possible LoF* on the basis of synaptic charge transfer were considered *Indeterminant.* Both of these two classifications may upon further study in a neuronal context turn out to be GoF or LoF. The term *Indeterminant* indicates appropriate caution when relying on parameters determined *in vitro* for recombinant receptors expressed in heterologous systems. Table 5 outlines these classification rules.

**Table 5:** Functional variant classification approach

**Likely LoF of GoF**	**Possible LoF or GoF**	**No detectable effect**	**Indeterminant**	Likely LoF(*)
One or more changes with high confidence and no conflicts in direction of change	Two or more changes with moderate confidence and no conflicts in direction of change One change of moderate confidence and >2.5-fold or <0.4-fold change in synaptic or non-synaptic charge transfer Conflicting changes of moderate/high confidence and >2.5-fold or <0.4-fold change in synaptic or non-synaptic charge transfer	No detectable functional changes for any parameters Only one change with moderate confidence and a change in synaptic and non-synaptic charge transfer between 0.4 and 2.5 fold	Conflicting changes in opposite functional direction and a change in synaptic and non-synaptic charge transfer between 0.4 and 2.5 fold	Large decrease in response amplitude that precludes other parameter assessments plus evidence for protein synthesis

Table 6 summarizes the net effect for *GRIN2B* variants evaluated in Tables 1 and 2 on predicted discrete parameters and charge transfer and then uses these rules to determine the variant classification as *Possible GoF*, *Likely GoF*, *Possible LoF*, *Likely LoF*, *Indeterminant* or *No Detectable Effect*. Four variants were GoF, one of which had conflicting results but was promoted by a strong increase in charge transfer (GluN2B-W607). Eight variants were LoF, two of which had conflicting results but were promoted based on strong reduction in the calculated synaptic charge transfer (GluN2B-M706V, GluN2B-V821F). Two variants had conflicting results and were classified as indeterminant (GluN2B-I150V, GluN2B-E657D). Comparison of the application of this approach to variants from patients and benign variants in tolerant domains from the general population (Table 6) validate this approach, as there are clearly no suprathreshold changes in discrete parameters for benign variants and none of the final calls are *Possible* or *Likely GoF* or *LoF*. In contrast, 86% of the patient-derived variants show detectable changes in functional parameters that leads to *Possible* or *Likely GoF* or *LoF.*

**Table 6:** Assessment of new *GRIN2B* variant-mediated fold changes in parameters supporting GoF and LoF

		**Relative functional effects**									**Final call**
***GRIN2B* variant**	**gnomAD alleles**	**Glutamate potency**	**Glycine potency**	**Mg** ^**2+**^ **IC**_**50**_	***P***_**OPEN**_	***τ***_**w**_	**Surface express.**	**Count** **(high, mod)**	**Synaptic charge transfer**	**Non-synaptic charge transfer**	**Classification**
**I150V**	0	$ \color{blue}{\text{H (5.7)}}$	$ \color{blue}{\text{M (1.5)}}$	0.74	$ \color{red}{\text{H (0.33)}}$	$ \color{blue}{\text{H (4.9)}}$	1.4	Conflict	1.1	1.7	Indeterminant
**S526P**	0	$ \color{red}{\text{M (0.50)}}$	1.1	0.85	1.0	tstm^a^	$ \color{red}{\text{H (0.10)}}$	1, 1	tstm^a^	$ \color{red}{\text{0.024}}$	$ \color{red}{\text{Likely LoF}}$
**G533D**	0	$ \color{red}{\text{H (0.12)}}$	$ \color{red}{\text{M (0.56)}}$	0.93	$ \color{red}{\text{M (0.57)}}$	$ \color{red}{\text{H (0.39)}}$	0.72	2, 2	$ \color{red}{\text{0.14}}$	$ \color{red}{\text{0.023}}$	$ \color{red}{\text{Likely LoF}}$
**W607S**	0	$ \color{red}{\text{M (0.78)}}$	$ \color{red}{\text{M (0.65)}}$	$ \color{blue}{\text{H (1729)}}$	$ \color{red}{\text{M (0.42)}}$	0.72	$ \color{red}{\text{M (0.62)}}$	Conflict	$ \color{blue}{\text{7.1}}$	$ \color{blue}{\text{7.2}}$	$ \color{blue}{\text{Possible GoF}}$
**E657D**	0	$ \color{blue}{\text{H (4.0)}}$	$ \color{blue}{\text{M (2.1)}}$	1.0	$ \color{red}{\text{H (0.40)}}$	$ \color{blue}{\text{H (3.5)}}$	1.0	Conflict	1.7	2.4	Indeterminant
**R682H**	0	$ \color{blue}{\text{M (1.6)}}$	$ \color{blue}{\text{M (1.7)}}$	$ \color{blue}{\text{M (1.6)}}$	1.2	$ \color{blue}{\text{M (1.5)}}$	1.3	$ \color{blue}{\text{0, 4}}$	$ \color{blue}{\text{3.2}}$	$ \color{blue}{\text{3.9}}$	$ \color{blue}{\text{Possible GoF}}$
**G689S**	0	$ \color{red}{\text{H (0.0002)}}$	$ \color{red}{\text{M (0.64)}}$	0.68	1.4	$ \color{red}{\text{H (0.011)}}$	0.78	$ \color{blue}{\text{2, 1}}$	$ \color{red}{\text{0.001}}$	$ \color{red}{\text{0.000012}}$	$ \color{red}{\text{Likely LoF}}$
**R693S**	0	$ \color{blue}{\text{H (14.2)}}$	1.1	0.69	$ \color{blue}{\text{M (1.5)}}$	$ \color{blue}{\text{H (7.8)}}$	0.69	2, 1	$ \color{blue}{\text{5.1}}$	$ \color{blue}{\text{10}}$	$ \color{blue}{\text{Likely GoF}}$
**I695T**	0	$ \color{red}{\text{H (0.16)}}$	1.1	0.75	$ \color{red}{\text{H (0.40)}}$	$ \color{red}{\text{M (0.50)}}$	$ \color{red}{\text{M (0.67)}}$	2, 2	$ \color{red}{\text{0.15}}$	$ \color{red}{\text{0.030}}$	$ \color{red}{\text{Likely LoF}}$
**I695S**	0	$ \color{red}{\text{H (0.01)}}$	1.3	1.0	$ \color{red}{\text{M (0.49)}}$	$ \color{red}{\text{H (0.36)}}$	$ \color{red}{\text{H (0.34)}}$	3, 1	$ \color{red}{\text{0.064}}$	$ \color{red}{\text{0.00043}}$	$ \color{red}{\text{Likely LoF}}$
**M706V**	0	$ \color{red}{\text{H (0.32)}}$	1.2	$ \color{blue}{\text{M (1.7)}}$	$ \color{red}{\text{M (0.65)}}$	0.69	$ \color{red}{\text{H (0.18)}}$	Conflict	$ \color{red}{\text{0.33}}$	$ \color{red}{\text{0.13}}$	$ \color{red}{\text{Possible LoF}}$
**E807K**	0	$ \color{red}{\text{M (0.40)}}$	0.7	$ \color{red}{\text{M (0.47)}}$	$ \color{red}{\text{H (0.37)}}$	$ \color{red}{\text{H (0.28)}}$	0.87	2, 2	$ \color{red}{\text{0.067}}$	$ \color{red}{\text{0.073}}$	$ \color{red}{\text{Likely LoF}}$
**S810N**	0	$ \color{blue}{\text{H (6.9)}}$	$ \color{blue}{\text{H (3.1)}}$	1.0	$ \color{blue}{\text{H (3.5)}}$	$ \color{blue}{\text{H (3.7)}}$	0.78	4, 0	$ \color{blue}{\text{8.2}}$	$ \color{blue}{\text{20}}$	$ \color{blue}{\text{Likely GoF}}$
**V821F**	0	$ \color{blue}{\text{M (1.6)}}$	$ \color{blue}{\text{M (2.1)}}$	0.81	$ \color{red}{\text{H (0.28)}}$	$ \color{red}{\text{H (0.12)}}$	1.2	Conflict	$ \color{red}{\text{0.059}}$	$ \color{red}{\text{0.87}}$	$ \color{red}{\text{Possible LoF}}$
**V21M**	**54**	0.95	0.88	0.70	1.1	0.88	1.0	Subthresh	0.67	0.71	No effect
**R84C**	**2**	0.83	1.1	0.90	1.4	1.0	$ \color{red}{\text{M (0.61)}}$	0, 1	1.2	0.89	No effect
**V172I**	**8**	1.0	1.1	1.0	1.3	0.71	0.95	Subthresh	0.85	1.1	No effect
**I227V**	**7**	1.0	1.4	0.85	1.0	1.0	1.3	Subthresh	0.82	0.88	No effect
**S383T**	**15**	1.0	$ \color{blue}{\text{M (1.8)}}$	1.2	0.80	1.0	1.1	0, 1	0.91	0.85	No effect
**W844R**	**5**	0.91	0.89	0.95	1.1	1.1	0.84	Subthresh	1.0	0.85	No effect
**A889T**	**5**	$ \color{blue}{\text{M (1.5)}}$	1.0	0.84	1.0	1.1	1.0	0, 1	1.2	1.8	No effect
**Y949H**	**2**	1.0	1.0	$ \color{red}{\text{M (0.58)}}$	0.91	1.0	0.78	0, 1	0.43	0.44	No effect
**P952L**	**2**	1.0	0.87	1.1	1.3	0.82	0.73	Subthresh	0.88	1.0	No effect
**G1026S**	**61**	1.0	1.1	1.2	1.0	0.87	0.67	Subthresh	0.63	0.73	No effect
**A1267S**	**68**	1.1	1.0	0.89	1.0	1.1	$ \color{red}{\text{M (0.62)}}$	0, 1	0.67	0.65	No effect
**G1369S**	**24**	1.0	1.0	1.2	1.0	0.92	0.95	Subthresh	0.86	0.89	No effect
**R1381Q**	**2**	0.82	1.0	1.4	1.0	0.81	0.91	Subthresh	0.82	0.79	No effect

Glutamate and glycine potency ratios are given as WT/variant EC_50_ because EC_50_ is reciprocally related to potency. Mg^2+^ IC_50_, open probability, weighted tau (*τ*_w_) and surface expression fold effects are given as variant/WT. A high (H) or moderate (M) confidence (see Table 4) for the change is indicated; red is LoF and blue is GoF. The number of high (H) and moderate (M) changes are given when there are no conflicts. Conflicting and subthreshold (indicated as Subthresh.) variants were promoted to *Possible GoF* or *Possible LoF* if the synaptic or non-synaptic charge transfer change was >2.5-fold or <0.4-fold. ^a^tstm—current responses were too small to measure functional endpoint precluding determination of *τ*_w_ and synaptic charge transfer, and consistent with LoF.

## Discussion

Here, we propose specific guidelines for classifying *GRIN* variant actions as GoF or LoF based on reproducible and comprehensive functional data obtained from validated, robust and widely accessible *in vitro* assays. We classify *GRIN* variants into six categories: *Likely GoF*, *Possible GoF*, *Likely LoF*, *Possible LoF*, *Indeterminant* and *No Detectable Effect*. We use a discrete classification method, which is similar to the approach used by ACMG to assess pathogenicity, as the primary means for determining GoF and LoF, with net synaptic and non-synaptic charge transfer as supporting information used to classify variants that are less clear. We discuss the rationale for selection of underlying criteria upon which to base these classifications, and consider multiple ways in which some of these assays can be accomplished. We validated this approach by recording and analyzing benign *GRIN2B* missense variants in tolerant regions of the receptor that uniformly show *No Detectable Effect*. We suggest that this classification system provides a first approximation of variant actions from highly accessible and scalable *in vitro* data. We employed strict statistical thresholds to increase patient homogeneity for clinical trials. However, it is important to note that some of the variants classified as *Indeterminant* or *No Effect* may, with additional clinical experience and advances in understanding of the basic science, in the future be reclassified as *Possible* or *Likely GoF/LoF.*

While ideally one would evaluate each variant in a neuronal context *in vivo*, this simply is not practical given the existence of many hundreds of known variants, with a likely upper limit in the thousands. It is not practical to construct this many different mutant mice or even neurons derived from induced pluripotent stem (iPS) cell lines and then evaluate them all with multiple assays, even though this might provide more definitive information. Thus, we submit that this approach relying on *in vitro* data provides a tractable working model from which to choose these designations, and thereby stratify variants. These designations can be revised for the variants later evaluated in animal models or neuronal systems. This approach could be extended to classify gene duplication or deletion as *Possible GoF* or *LoF*. In addition, the approach is generalizable for other synaptic ion channels and any protein for which multiple functional readouts are available.

To further illustrate the utility of this approach, we have applied it to published variants for which a full data set is available (Supplementary Material, Tables S1–S3), and to published variants that required assessment of one additional parameter to complete the data set (Supplementary Material, Table S4). In addition, current responses for 10 published missense variants were too small to be detected, and thus prevented accurate estimation of the variants’ pharmacological (i.e. agonist potency and sensitivity to Mg^2+^) and biophysical (i.e. channel open probability and deactivation time course) properties (21,26,27,31) (Supplementary Material, Table S5). When the reduction in response amplitude is so great that experimental assessment of variant effects on parameters is not determinable, missense variants are classified as Likely LoF^*^ without complete characterization (see rules in Table 5). This allows us to categorize the 49 published patient-derived variants for which a full dataset exists (see Supplementary Material, Tables S3–S5). *Likely GoF* published variants include GluN1-R548Q, GluN1-L551P, GluN2A-E551K, GluN2A-S554T, GluN2A-L611Q, GluN2B-S555N and GluN2B-R969H. *Possible GoF* published variants include GluN1-Q559R, GluN1-M641I, GluN2A-V506A, GluN2A-P552R, GluN2A-N615K, GluN2B-S541G, GluN2B-W607C, GluN2B-G611V, GluN2B-N615I, GluN2B-N615K, GluN2B-N616K, GluN2B-V620M and GluN2D-L670F. *Likely LoF* published variants include GluN1-P532H, GluN1-S549R, GluN2A-G483R, GluN2A-A716T, GluN2A-D731N, GluN2B-E413G, GluN2B-S541R, GluN2B-P553T, GluN2D-S573F and GluN2D-R1313W. *Possible LoF* variants include GluN2A-S545L, GluN2A-M705V, GluN2A-V734L, GluN2B-C461F and GluN2D-S1271L. In addition, we have classified variants with expression too low to measure responses (GluN2A-R518H, GluN2A-T531M, GluN2A-A548P, GluN2B-C436R, GluN2B-A549V, GluN2B-F550S, GluN2B-L551S, GluN2B-S555I, GluN2B-P553L, GluN2B-A636P) as Likely LoF. Six published variants had *No Effect* or were *Indeterminant* (GluN2A-R504W, GluN2A-K669N, GluN2A-P699S, GluN2B-R540H).

We recognize that the designation of GoF and LoF can be simplistic and may not be satisfying to those working at high levels of resolution on receptor properties, receptor structure, circuit function or clinical implications. However, collection of the comprehensive data set needed to make these determinations will ultimately allow more detailed analysis and subdivision as more variants are identified and patients enter clinical research registries, and this approach is critically needed in order to move forward with clinical trials. Therefore, this strategy serves multiple purposes. These data will also help suggest which variants to study in mouse models. The approach here mirrors what is currently regarded as ‘minimal clinically important difference’, which is a concept used by regulatory agencies when assessing effectiveness of clinical interventions. There is some variability in how this is determined in clinical outcome measures, and in some cases it is 0.5 ^*^ SD (60), which is below our thresholds. Implementation of this approach will create data for future detailed analysis of function at a higher resolution, while at the same time providing invaluable classification as clinical trials are designed with pharmacological and future genetic approaches. This will then link magnitude of GoF or LoF with diagnostic severity, precision therapeutic approaches and meaningful clinical improvements in affected individuals.

## Materials and Methods

### Source of variants and molecular biology

All variants for which new data were obtained are in the public domain, and described in ClinVar or the peer-reviewed literature. Recombinant cDNA utilized corresponded to human GluN1-1a (referred to as GluN1; NCBI Reference Sequence NM_007327.3), GluN2A (NM_000833.4) and GluN2B (NM_000834.4) in the plasmid pcIneo. Mutagenesis was performed on cDNA using the QuikChange protocol (Stratagene La Jolla, CA, USA). Sequences were verified using dideoxy DNA sequencing (Eurofins Genomics, Louisville, KY, USA). The cDNA was linearized using FastDigest (ThermoFisher, Waltham, MA) restriction digestion at 37°C for 1 h. Complementary RNA (cRNA) was synthesized *in vitro* from linearized WT and variant cDNA using the mMessage mMachine T7 kit (Ambion, Austin, TX, USA).

### Two-electrode voltage clamp recordings from oocytes

Stage V–VI *Xenopus laevis* oocytes were obtained from commercial vendors as previously described (21,25,26,28,29,31,42,43,49). Briefly, we prepared unfertilized *X. laevis* oocytes from commercially obtained ovaries (Xenopus One, Inc.), which were digested with Collagenase Type 4 (Worthington-Biochem, Lakewood, NJ, USA) at a concentration of 800 μg/ml in Ca^2+^-free Barth’s solution, which contained (in mm) 88 NaCl, 2.4 NaHCO_3_, 1 KCl, 0.82 MgSO_4_ and 10 HEPES (pH 7.4 with NaOH), supplemented with 100 μg/ml gentamycin, 1 U/ml penicillin and 1 μg/ml streptomycin (15 ml for a half ovary). The ovary was incubated in enzyme solution with gentle mixing at 23°C for 2 h. Oocytes were rinsed 10 times with Ca^2+^-free Barth’s solution (35–40 ml each time) for 5 min and rinsed 4 more times with normal Barth’s solution (i.e. including 0.41 mm CaCl_2_ and 0.33 mm Ca(NO_3_)_2_) on a slow shaker. Oocytes were injected with cRNA encoding either WT or variant NMDAR subunits (GluN1:GluN2 ratio 1:2). Total weight of cRNA was 0.25–10 ng in 50 nl of RNAase-free water per oocyte; in rare cases for variant NMDARs that express at low levels, RNA was increased to 25 ng/oocyte. Oocytes were maintained in normal Barth’s solution at 16°C. Two-electrode voltage clamp recordings were performed at 23°C as previously described. A dual-stage micropipette puller was used to prepare the microelectrodes from borosilicate glass with resistance of 4–8 MΩ (TW150F-4; World Precision Instruments, Sarasota, FL). Current and voltage electrodes were filled with 0.3 or 3 M KCl, respectively, and used for recordings of oocytes 1–5 days after injection. Oocytes were placed in a multi-track recording chamber that shared a single perfusion line, allowing simultaneous recordings. Oocytes expressing recombinant NMDARs were perfused with solution containing (in mm) 90 NaCl, 1.0 KCl, 0.5 BaCl_2_, 10 HEPES and 0.01 EDTA adjusted to pH 7.4 with NaOH. For Mg^2+^ potency studies, EDTA was omitted from the recording solution.

### l-Glutamate potency assay

The oocyte membrane potential was held under voltage clamp at −40 mV, and oocytes were superfused with buffer including sequentially increasing concentrations of l-glutamate (6–7 concentrations) for 0.75 min duration each in the continuous presence of 100 μm glycine to obtain concentration-response data. l-Glutamate concentrations were selected to achieve maximal activation at the highest concentration. If variants studied reduced the glycine potency, the concentration of glycine was increased to at least 10 times higher than the variant glycine EC_50_. Results at each l-glutamate concentration are normalized to the maximum receptor activation levels achieved (defined as 100%). The data are fitted to obtain an EC_50_ value for each oocyte by


(1) \begin{equation*} Response\ \left(\%\right)=100/\left(1+{\left({EC}_{50}/\left[ agonist\right]\right)}^{nH}\right), \end{equation*}

where *EC_50_* is the agonist concentration that elicited a half maximal response and *nH* is the Hill slope.

### Glycine potency assay

The oocyte membrane potential was held under voltage clamp at −40 mV and oocytes were superfused with buffer including sequentially increasing concentrations of glycine for 0.75 min duration each (6–7 concentrations) in the continuous presence of 100 μm l-glutamate to obtain concentration-response data. Glycine concentrations were selected to achieve near maximal activation at the highest concentration. If the variant reduced l-glutamate potency, the l-glutamate concentration was increased to a value at least 10 times the variant l-glutamate EC_50_. Results at each glycine concentration were normalized to the maximum receptor activation levels achieved (defined as 100%). The data were fitted by Equation 1 to obtain an EC_50_ value for each oocyte.

### Mg^2+^ potency assay

The oocyte membrane potential was held under voltage clamp at −40 mV to −60 mV. After a steady baseline was obtained, oocytes were activated by the application of maximally effective concentrations of l-glutamate and glycine. If the variant reduced potency of either co-agonist, l-glutamate and glycine concentrations were increased to at least 10 times the EC_50_ value. Following challenge with a maximally effective concentration of agonists, increasing concentrations of Mg^2+^ (concentration varies depending on the specific receptor and variant tested) were co-applied in the continuous presence of maximal l-glutamate and glycine. Results at each Mg^2+^ concentration were normalized to the maximum receptor activation levels without Mg^2+^ (defined as 100%) and IC_50_ values obtained by fitting concentration–inhibition data with


(2) \begin{align*}& Response\ \left(\%\right)\notag\\ &=\left(100- minimum\right)/\left(1+{\left(\left[{\mathrm{Mg}}^{2+}\right]/{IC}_{50}\right)}^{nH}\right)\ + minimum, \end{align*}

where *minimum* is the residual percent response in saturating concentration (constrained to be >0) of Mg^2+^, *IC_50_* is the concentration of Mg^2+^ that causes half maximal inhibition and *nH* is the Hill slope. Inhibition at 1 mm Mg^2+^ will be directly taken from the data or calculated from fitted *IC_50_* and *nH*.

### Open probability assay

Variant GluN1 subunits were co-expressed with GluN2A-A650C or GluN2B-A651C and variant GluN2 subunits were expressed with GluN1-A652C cRNA at the same ratios as described before (61,62). The oocyte membrane potential was held under voltage clamp at −40 mV and oocytes superfused with buffer including a maximally effective concentration of l-glutamate and glycine for 1 min duration. If the variant reduces l-glutamate or glycine potency, the concentrations were increased to values at least 10 times the EC_50_. The solution was then switched for at least 3 min to one in which maximally effective concentrations of agonists are supplemented with 0.2 mm of the covalent modifying reagent 2-aminoethyl methanethiolsulfonate hydrobromide (MTSEA; Toronto Research Chemicals, Ontario, Canada), which was prepared fresh and used within 30 min. The channel open probability was estimated from the fold potentiation observed in MTSEA according to


 (3) \begin{equation*} Open\ probability=\left({\gamma}_{\mathrm{MTSEA}}/{\gamma}_{\mathrm{CONTROL}}\ \right)\times \left(1/ Potentiation\right), \end{equation*}

where *γ*_MTSEA_ and *γ*_CONTROL_ were the single-channel chord conductance values estimated from GluN1/GluN2A receptors and *Potentiation* was defined as the ratio of current in the presence of MTSEA to current in the absence of MTSEA; *γ*_MTSEA_ / *γ*_*CONTROL*_ was 0.67 (62). Variants residing within one helical turn of the sites MTSEA modifies (GluN1-A652, GluN2A-A650, GluN2B-A651) might alter this assay and results for these few variants should be interpreted cautiously.

### Deactivation time course assay

We determined the deactivation time course as previously described (26). Briefly, transfection of cDNA into mammalian cells was accomplished by the Ca^2+^ phosphate method (63). For our determination of the deactivation time constant, human embryonic kidney (HEK) 293 cells (ATCC CRL-1573) were plated on 12 mm glass coverslips pretreated with 0.1 mg/ml poly-d-lysine and placed into 12-well plates with Dulbecco’s modified Eagle medium (Gibco 10569-010, DMEM + GlutaMAX) supplemented with 10% dialyzed fetal bovine serum and 10 U/ml penicillin and 10 μg/ml streptomycin. HEK cells were maintained at 37°C in a humidified environment with 5% CO_2_. The cells were transiently transfected with cDNA (total 0.5 mg/well) encoding human GluN1, GluN2A and eGFP at a ratio of 1:1:5 or GluN1, GluN2B and eGFP at a ratio of 1:1:1 by the calcium phosphate method (63). NMDAR antagonists (200 μm DL-APV and 200 μm 7-CKA) were added after the transfection. After 12–24 h following transfection, the cells were transferred to a recording chamber and perfused with external recording solution that contained (in mm) 3 KCl, 150 NaCl, 0.01 EDTA, 1.0 CaCl_2_, 10 HEPES and 22 d-mannitol (adjusted to pH 7.4 with NaOH). The patch electrodes (resistance 3–5 MΩ) were prepared from thin-walled glass micropipettes (TW150F-4; World Precision Instruments, Sarasota, FL, USA) with the use of a dual-stage micropipette puller and filled with internal solution containing (in mm) 110 d-gluconate, 110 CsOH, 30 CsCl, 5 HEPES, 4 NaCl, 0.5 CaCl_2_, 2 MgCl_2_, 5 BAPTA, 2 NaATP and 0.3 NaGTP (pH 7.4 with CsOH; 300–305 mOsmol/kg). Whole-cell current responses were evoked by application of maximally effective concentrations of agonists (1 mm glutamate and 100 μm glycine) at a holding potential of −60 mV and recorded by an Axopatch 200B or Warner PC505B amplifier. If a variant reduces potency of either co-agonist, concentrations were increased to at least 10 times the EC_50_ value. All whole-cell recordings were performed at 23°C. The current responses were low-pass filtered at 8 kHz with an 8-pole Bessel filter (−3 dB; Frequency Devices) and digitized at 20 kHz using a Digidata 1440A acquisition system (Molecular Devices) controlled by Clampex 10.3 (Molecular Devices). The position of a two-barreled theta-glass micropipette used for rapid solution exchange was controlled by a piezoelectric translator (Burleigh Instruments, Newton, NJ, USA or Siskiyou, Grants Pass, OR, USA) such that the cell was exposed to agonist for either 2–8 ms or for 1 s. Data were filtered off-line at 2 kHz, and the deactivation time course fitted by a dual exponential function


(4) \begin{align*} Response=& {Amplitude}_{FAST}\ \left(\exp \left(- time/{tau}_{FAST}\right)\right)\notag\\&+{Amplitude}_{SLOW}\ \left(\exp \left(- time/{tau}_{SLOW}\right)\right). \end{align*}

The weighted deactivation tau *τ*_w_ was calculated by


(5) \begin{align*} {\tau}_{\mathrm{w}}=\left({Amplitude}_{FAST}\ {tau}_{FAST}+{Amplitude}_{SLOW}\ {tau}_{SLOW}\ \right)\notag/\\\left({Amplitude}_{FAST}+{Amplitude}_{SLOW}\right). \end{align*}

Current responses for some cells with larger amplitudes were corrected for series resistance filtering off line (64) prior to fitting.

### Surface protein assay

For determination of surface expression, we assayed beta-lactamase (β-lac) activity in cells transfected with NMDAR variant subunits that were fused in-frame at the end of the signal peptide sequence for GluN1, GluN2A or GluN2B subunits to the β-lac-open reading frame so that active β-lac enzyme faced the extracellular solution (21,25,31). HEK cells were plated in 96-well plates at a density of 50 000 cells/well in serum-supplemented DMEM. HEK cells in 96-well plates were transiently transfected 24 h after plating with cDNA encoding β-lac-GluN1 variants with WT GluN2, or β-lac-GluN2 variants with WT GluN1 using Fugene6 according to manufacturer’s instructions (Promega, Madison, WI). Several wells in each plate were treated with Fugene6 alone without cDNA to define background absorbance. Six wells were transfected for each condition to allow determination of surface and total protein levels in three wells each. NMDAR antagonists (200 μm DL-APV and 200 μm 7-CKA) were added at the time of transfection. After 24 h, cells were rinsed with Hank’s Balanced Salt Solution (HBSS) containing (in mm) 140 NaCl, 5 KCl, 0.3 Na_2_HPO_4_, 0.4 KH_2_PO_4_, 6 glucose, 4 NaHCO_3_ and supplemented with 10 mm HEPES (pH 7.4). Subsequently, 100 μl of a 100 μm nitrocefin (Millipore, Burlington, MA, USA) solution in HBSS with HEPES was added to each of the three wells for measuring the level of extracellular enzymatic activity, which reflected NMDAR surface expression. The other three wells were lysed by incubation in 50 μl H_2_O for 30 min prior to the addition of 50 μl of 200 μm nitrocefin to determine the level of total enzymatic activity, which reflects the total NMDAR subunit protein expression level. The absorbance at 486 nm was determined using a microplate reader every min for 30 min at 30°C. The rate of increase in absorbance was generated from the slope of a linear fit to the data.

### Synaptic and non-synaptic charge transfer

We estimated synaptic charge transfer from *in vitro* data obtained in heterologous expression systems for recombinant receptors, as previously described (21,25,29,31). The effects of a *GRIN* variant on the relative synaptic charge transfer can be estimated according to


\begin{align*} Relative\ synaptic\ {charge\ transfer}_{\mathrm{Variant}/\mathrm{WT}}\\={\tau}_{\mathrm{w},\mathrm{Variant}}/{\tau}_{\mathrm{w},\mathrm{WT}}\times{P}_{\mathrm{Variant}}/{P}_{\mathrm{WT}}\times \end{align*}

(6) \begin{equation*} {Surface}_{\mathrm{Variant}}/{Surface}_{\mathrm{WT}}\times{R}_{\mathrm{GLYCINE}}\times{R}_{\mathrm{GLUTAMATE}}\times{\text{Mg}}_{\mathrm{Variant}}/{\text{Mg}}_{\mathrm{WT}} \end{equation*}

where each ratio is the fold effect of the variant compared with WT for weighted tau (*τ*_w_), open probability (*P*), surface expression (*Surface*) and degree of current recorded at −60 mV in the presence of 1 mm Mg^2+^ (Mg). The relative receptor response (*R*) predicted for 1 mm synaptic glutamate and 3 μm glycine is calculated from the fitted values for glutamate and glycine EC_50_ and Hill slope (nH) according to Equation 1. *R*_AGONIST_ shown is the predicted response for variant divided by predicted response for WT receptor (where EC_50_ units are mol/L),


(7) \begin{align*} {R}_{\mathrm{GLUTAMATE}}=\left(1+{\left({EC}_{50WT}/0.001\right)}^{nHWT}\right)\notag/\\\left(1+{\left({EC}_{50Variant}/0.001\right)}^{nHVariant}\right) \end{align*}

and


(8) \begin{align*} {R}_{\mathrm{GLYCINE}}=\left(1+{\left({EC}_{50WT}/0.000003\right)}^{nHWT}\right)\notag/\\\left(1+{\left({EC}_{50Variant}/0.000003\right)}^{nHVariant}\right). \end{align*}

We recognize that the EC_50_ value determined from steady-state glutamate exposure overestimates potency to brief exposure to 1 mm glutamate within synapses (66). Thus, *R*_GLUTAMATE_ determined with the steady-state EC_50_ and 1 mm glutamate will underestimate potential effects of variant-induced changes in EC_50_. To further capture variant-induced changes in EC_50_, we calculated the non-synaptic charge transfer, which is relevant for spillover of glutamate onto perisynaptic receptors as well as for NMDARs activated by tonic glutamate present outside the synapse or released from glial cells. For non-synaptic charge transfer we omitted *τ*_w,Variant_/*τ*_w,WT_ from Equation 6 and use the following equation to determine relative response as a function of glutamate concentration, given the low concentration of extrasynaptic glutamate of less than 100 nm (55–58):


(9) \begin{align*} {R}_{\mathrm{GLUTAMATE}}=\left(1+{\left({\mathrm{EC}}_{50\mathrm{WT}}/0.0000001\right)}^{\mathrm{nHWT}}\right)\notag/\\\left(1+{\left({\mathrm{EC}}_{50\mathrm{Variant}}/0.0000001\right)}^{\mathrm{nHVariant}}\right). \end{align*}

*Conflict of interest statement.* S.F.T. is a member of the SAB for Eumentis Therapeutics, Sage Therapeutics and Combined Brain, is a member of the Medical Advisory Board for the GRIN2B Foundation and the CureGRIN Foundation, is an advisor to GRIN Therapeutics and Neurocrine, is co-founder of NeurOp Inc. and Agrithera Inc., and is a member of the Board of Directors of NeurOp Inc. H.Y. is PI on a research grant from Sage Therapeutics to Emory University School of Medicine. S.J.M. is PI on a research grant from GRIN Therapeutics to Emory University School of Medicine. T.A.B. received research funding from the International Foundation for CDKL5 Research, Loulou Foundation and the National Institutes of Health; consultancy for Alcyone, AveXis, GRIN Therapeutics, GW Pharmaceuticals, the International Rett Syndrome Foundation, Marinus Pharmaceuticals, Neurogene, Ovid Therapeutics and Takeda Pharmaceutical Company Limited; clinical trials with Acadia Pharmaceuticals Inc., GW Pharmaceuticals, Marinus Pharmaceuticals, Ovid Therapeutics and Rett Syndrome Research Trust; all remuneration has been made to his department. J.M.B is a member of the SAB for Combined Brain, Yellow Brick Road Project and GRIN2B Foundation; advisor to GRIN Therapeutics; received research funding from Ovid Therapeutics and Simons Searchlight.

## Supplementary Material

Supplemental_Information_8-7-23_FINAL6_ddad104

## Data Availability

The published article includes all datasets/code generated or analyzed during this study.
