# Supplementary material for: Classification of missense variants in the N-methyl-d-aspartate receptor GRIN gene family as gain- or loss-of-function
Source: Hum Mol Genet. 2023 Jun 27;32(19):2857–71. doi: 10.1093/hmg/ddad104 (PMC10508039; doi:10.1093/hmg/ddad104)
Supplement: Supplemental_Information_8-7-23_FINAL6_ddad104 [file supplemental_information_8-7-23_final6_ddad104.pdf]

SUPPLEMENTAL INFORMATION

Classification of missense variants in the N-methyl-D-aspartate receptor GRIN gene family as gain- or loss-of-function

Scott J. Myers[1,2,*], Hongjie Yuan[1,2,*], Riley E. Perszyk[1], Jing Zhang[1], Sukhan Kim[1], Kelsey A. Nocilla[1], James P. Allen[1,2], Jennifer M. Bain[3], Johannes R. Lemke[4,5], Dennis Lal[6,7,8,9], Timothy A. Benke[10], Stephen F. Traynelis[1,2,11]

[1] Department of Pharmacology and Chemical Biology, Emory University School of Medicine, Atlanta, GA 30322, USA

[2] The Center for Functional Evaluation of Rare Variants (CFERV), Emory University School of Medicine, Atlanta, GA 30322, USA

[3] Department of Neurology, Division of Child Neurology, Columbia University Irving Medical Center, New York, NY 10032, USA

[4] Institute of Human Genetics, University of Leipzig Medical Center, Leipzig 04103, Germany

[5] Center for Rare Diseases, University of Leipzig Medical Center, Leipzig 04103, Germany

[6] Genomic Medicine Institute, Lerner Research Institute, Cleveland Clinic, Cleveland, OH 44106, USA

[7] Epilepsy Center, Neurological Institute, Cleveland Clinic, Cleveland, OH 44195, USA

[8] Stanley Center for Psychiatric Research, Broad Institute of MIT and Harvard, Cambridge, MA 02142, USA

[9] Cologne Center for Genomics (CCG), Medical Faculty of the University of Cologne, Koln 50923, Germany

[10] Department of Pediatrics and Neurology, University of Colorado School of Medicine, and Children's Hospital Colorado, Aurora, CO 80045, USA

[11] Emory Neurodegenerative Disease Center, Atlanta, GA 30322, USA

<u>**Table of Contents**</u>

Additional Assay Protocols that Allow Future Analysis of Variant Properties

Supplemental Table S1. Number of variants described in the literature that were evaluated in this study

Supplemental Table S2. Predicted synaptic and non-synaptic charge transfer changes relative to WT NMDARs

Supplemental Table S3. Published variant-mediated changes in parameters supporting GoF and LoF status

Supplemental Table S4. New data recorded to supplement published data

Supplemental Table S5. Summary of *GRIN* variants with no detectable current response in both *Xenopus* oocytes and transfected HEK cells

Supplemental Figure S1. The relationship between log(fold shift in glutamate EC_{50}) and log (fold shift in tau deactivation)

Additional Assay Protocols that Allow Future Analysis of Variant Properties

We have previously published analysis of pH and Zn^{2+} inhibition of NMDA receptors (see https://grin-portal.broadinstitute.org/) using standard protocols. We suggest that it would be helpful to determine these parameters for variant NMDA receptors to obtain additional data that could be used in the future to further stratify patients and explore correlations in receptor function and clinical phenotype. We suggest that future experiments follow the methods previously reported to ensure data is harmonized with that already published (e.g. Yuan et al., 2014; Ogden et al., 2017; Chen et al., 2017; Xie et al., 2023). Below are proposed assay methods for these additional parameters.

Zn^{2+} Assay

Xenopus laevis oocytes were prepared, maintained, and injected with cRNA encoding human NMDA receptor subunits as described in the Methods. Oocytes expressing recombinant human NMDA receptors were perfused with solution containing (in mM) 90 NaCl, 1 KCl, 0.5 $BaCl_2$, 10 Tricine, and 10 HEPES adjusted to pH 7.3 (at 23°C) with NaOH. The oocyte membrane potential was held under voltage clamp at -20 mV. After a steady baseline was obtained, oocytes were maximally activated with 50 μM L-glutamate and 50 μM glycine, and then in the continuous presence of maximal L-glutamate and glycine were superfused with increasing concentrations of Zn^{2+}; the concentration varied depending on the specific receptor and variant tested. If the variant reduced agonist potency, the concentration of L-glutamate and/or glycine were increased to be at least 10 times the EC_{50} values. To achieve low concentrations of extracellular Zn^{2+}, $ZnCl_2$ was added to the extracellular recording solution, which contained 10 mM of the Zn^{2+} buffer tricine. We used a pKa for tricine of 8.15 to calculate the concentration of ionized tricine at pH 7.3 capable of binding Zn^{2+}. The following buffered free Zn^{2+} concentrations were achieved by adding nominal concentrations of $ZnCl_2$ to tricine-containing external oocyte recording solution: for 1 nM free Zn^{2+} 0.14 μM $ZnCl_2$ was added, for 3 nM free Zn^{2+} 0.42 μM $ZnCl_2$ was added, for 10 nM free Zn^{2+} 1.4 μM $ZnCl_2$ was added, for 30 nM free Zn^{2+} 4.2 μM $ZnCl_2$ was added, for 100 nM free Zn^{2+} 14 μM $ZnCl_2$ was added, for 300 nM free Zn^{2+} 42 μM $ZnCl_2$ was added (see Traynelis et al., 1998 for equations). If the variant reduced Zn^{2+} sensitivity, higher concentrations were tested. $ZnCl_2$ was made up fresh

immediately before addition to tricine-containing solution. Response amplitudes at each buffered Zn^{2+} concentration should be normalized to the maximum receptor activation levels without Zn^{2+} (defined as 100%) and IC_{50} values obtained by fitting concentration-inhibition data with Equation 2 in the Methods, which was

$$Response\ (\%) = (100 - minimum) / (1 + ([concentration\ Zn^{2+}] / IC_{50})^{nH}) + minimum,$$

where *minimum* is the residual percent response in saturating concentration (constrained to be > 0) of Zn^{2+}, IC_{50} is the concentration of Zn^{2+} that causes half maximal inhibition, and *nH* is the Hill slope. Statistical comparison between WT and variant receptor results should be made using a two-tailed unpaired t-test on the log of the fitted IC_{50} values or directly on the fitted *minimum* current in saturating concentrations of Zn^{2+}.

pH Assay

Xenopus laevis oocytes were prepared, maintained, and injected with cRNA encoding human NMDA receptor subunits as described in the Methods. *Xenopus* oocytes expressing recombinant human NMDA receptors were perfused with solution containing (in mM) 90 NaCl, 1 KCl, 0.5 $BaCl_2$, 10 HEPES, and 0.01 EDTA, adjusted to pH 7.6 with NaOH, and then a portion of this solution adjusted to pH 6.8 with HCl. The order of pH adjustment is important to ensure the same concentration of the permeant ion Na^+ is present at both pH values. The oocyte membrane potential should be held under voltage clamp at -40 mV. After a steady baseline was obtained in pH 7.6 recording buffer, the oocytes should be activated with maximally effective concentration of co-agonists (100 µM L-glutamate and 100 µM glycine) in pH 7.6 buffer. If the variant reduced agonist potency, the concentration of agonists should be increased so that their concentration is at least 10 times the EC_{50} value. Following a washout period, the oocytes should be washed in pH 6.8 buffer, and then maximally activated with 100 µM L-glutamate and 100 µM glycine in pH 6.8 buffer. The current at pH 6.8 is then compared to the current at pH 7.6 (defined as 100%). All recordings should be made at 23°C. Statistical comparison between wild type and variant receptor results should be made using a two-tailed unpaired t-test.

Supplemental Table S1. Number of variants described in the literature that were evaluated in this study

Number of GRIN variants	Source (PMID)
4	35110392
14	28377535
1	24504326, 24839611
3	28283559
1	24863970, 27839871, 30168177
7	27616483, 31504254
25	24863970, 27839871, 28182669, 30168177
6	28095420
1	28126851
10	24839611, 31429998
1	29644724
5	29365063
1	32577763
1	34227748
1	34413877
10	37000222
1	28377535
1	28377535; 31429998
2	35393335
1	28377535, 35393335
2	35983985
1	30168177
**100**	**Total**

Supplemental Table S2. Predicted synaptic and non-synaptic charge transfer changes relative to WT NMDARs

GRIN Variants	Parameters of Variant Actions on Receptor Function							Non-Synaptic Function			Source
	$R_{GLU,SYNAPSE}$	R_{GLY}	1 mM Mg^{2+} -60 mV	P_{OPEN}	Tau_W	Surface expression	Charge Transfer	$R_{GLU,NONSYNAPSE}$	Charge Transfer	Promote?	PMID
**1-P532H**	0.96	0.92	2.2	0.42	0.42	0.64	0.22	0.01	0.004	Promotes	3441387
**1-R548Q**	1.0	1.1	0.63	2.0	2.8	1.2	4.5	3.7	5.9	Promotes	3700022
**1-S549R**	1.0	0.65	1.3	0.08	0.63	1.3	0.06	0.35	0.03	Promotes	3700022
**1-L551P**	1.0	1.2	0.71	0.88	7.3	0.75	4.2	30	17	Promotes	2936506
**1-Q559R**	1.0	0.92	0.57	1.9	1.9	1.6	2.9	1.6	2.5	Promotes	3700022
**1-M641I**	1.0	1.3	9.4	0.35	1.2	0.71	3.6	0.76	2.2	Promotes	3422774
**2A-G483R**	0.98	0.92	0.39	0.87	0.36	0.57	0.06	0.03	0.006	Promotes	2783987
**2A-R504W**	1.0	1.1	1.4	1.1	1.6	0.36	0.91	2.2	1.3	--	2783987
**2A-V506A**	1.0	1.1	2.6	1.1	1.4	1.1	4.8	2.8	9.1	Promotes	2783987
**2A-S545L**	1.0	0.94	1.2	0.52	0.94	0.89	0.51	0.61	0.34	Promotes	3700022
**2A-E551K**	1.0	1.2	0.97	2.0	3.0	0.68	4.9	12	19	Promotes	3700022
**2A-P552R**	1.0	1.2	0.52	1.2	15	1.3	15	27	26	Promotes	2809542
**2A-S554T**	1.0	1.2	0.63	4.1	3.2	1.4	14	10	43	Promotes	3700022
**2A-L611Q**	1.0	1.2	23	1.2	2.3	0.73	55	1.7	41	Promotes	3142999
**2A-N615K**	1.0	1.1	36	0.67	1.2	0.74	24	2.1	41	Promotes	3142999
**2A-K669N**	1.0	0.87	0.79	0.22	4.3	0.97	0.63	11	1.7	--	2783987
**2A-P699S**	1.0	0.98	1.0	0.89	0.93	0.83	0.70	3.1	2.3	--	2783987
**2A-M705V**	1.0	1.1	1.2	0.41	1.0	0.68	0.35	1.1	0.38	Promotes	2783987
**2A-A716T**	0.95	0.97	0.94	0.89	0.57	0.40	0.18	2.0	0.65	Promotes	2783987
**2A-D731N**	0.06	0.85	0.54	0.21	0.25	0.22	0.0003	$6x10^{-6}$	$1x10^{-7}$	Promotes	2783987
**2A-V734L**	1.0	1.0	1.1	0.90	0.54	0.95	0.52	0.24	0.23	Promotes	2783987
**2B-E413G**	0.97	1.0	1.4	1.0	0.04	0.18	0.008	0.003	0.001	Promotes	2486397
**2B-C461F**	0.92	1.0	1.2	1.0	0.05	0.16	0.009	0.002	0.0004	Promotes	2783987
**2B-S541G**	1.0	0.98	0.74	1.8	2.3	0.6	1.8	5.7	4.4	Promotes	3700022
**2B-S541R**	1.0	0.84	0.38	0.54	0.13	1.1	0.03	0.02	0.004	Promotes	3700022
**2B-R540H**	1.0	1.0	0.57	2.0	2.0	0.43	1.0	3.2	1.6	--	2783987
**2B-P553T**	1.0	0.80	0.59	1.0	0.19	1.2	0.11	0.52	0.29	Promotes	3700022
**2B-S555N**	1.0	1.0	1.3	1.8	2.3	0.97	4.9	6.9	15	Promotes	3700022
**2B-W607C**	1.0	1.0	42	0.32	0.61	0.84	7.0	0.52	5.9	Promotes	3142999
**2B-G611V**	1.0	0.97	29	0.54	1.5	1.2	28	0.65	12	Promotes	2837753
**2B-N615I**	1.0	1.1	43	0.54	0.88	0.79	17	1.4	27	Promotes	3142999
**2B-N615K**	1.0	1.0	60	0.54	1.2	0.53	21	1.5	27	Promotes	2837753
**2B-N616K**	1.0	1.1	63	0.54	1.3	1.1	50	0.93	37	Promotes	2837753
**2B-V620M**	1.0	0.95	6.4	1.5	1.6	0.58	8.6	1.9	9.9	Promotes	3142999
**2B-R696H**	1.0	0.99	0.58	1.0	3.6	0.75	1.5	11	4.8	Promotes	2783987
**2D-S573F**	1.0	1.0	0.42	0.24	0.73	0.60	0.04	1.8	0.11	Promotes	3150425
**2D-L670F**	1.0	1.0	0.66	54	6.7	0.62	148	3.1	69	Promotes	3150425
**2D-S1271L**	1.0	0.98	1.3	0.48	0.91	0.69	0.37	1.8	0.73	Promotes	3150425
**2D-R1313W**	1.0	0.98	1.1	0.37	0.89	0.33	0.12	1.3	0.18	Promotes	3150425

The relative synaptic charge transfer was calculated using equations 6-8 (*see* Methods) and non-synaptic charge transfer was calculated using equations 6,8,9 as described in the main text (see Swanger at al. 2016, Li et al. 2019, Xu et al. 2021, Xie et al. 2023). The threshold for promoting classification to *Possible GoF* or *Possible LoF* was a relative fold change in synaptic or non-synaptic charge transfer that was greater 2.5 or less than 0.4, respectively. Potential promotion (if needed, see Table 5) of GoF fold values are blue and LoF fold values are red.

Supplemental Table S3. Published variant-mediated changes in parameters supporting GoF and LoF status

	Relative Functional effects								Final Call
GRIN-Variant	Glutamate Potency	Glycine Potency	Mg^{2+} IC_{50}	P_{OPEN}	τ_W	Surface Express.	Count (High, Mod)	Charge Transfer[a]	Classification
1-P532H	H (0.06)	0.90	0.86	H (0.42)	H (0.42)	M (0.64)	3,1	0.22, 0.004	Likely LoF
1-R548Q	H (3.3)	M (1.8)	1.3	H (2.0)	H (2.8)	1.2	3,1	4.5, 5.9	Likely GoF
1-S549R	M (0.51)	H (0.40)	1.1	H (0.08)	M (0.63)	1.3	2,2	0.06, 0.03	Likely LoF
1-L551P	H (20)	H (109)	0.91	0.88	H (7.3)	0.75	3,0	4.2, 17	Likely GoF
1-Q559R	1.3	0.86	1.0	M (1.9)	M (1.9)	M (1.6)	0,3	2.9, 2.5	Possible GoF
1-M641I	1.1	M (1.7)	H (8.1)	H (0.35)	1.2	0.71	Conflict	3.6, 2.2	Possible GoF
2A-G483R	H (0.06)	0.80	1.0	0.87	H (0.36)	M (0.57)	2,1	0.06, 0.006	Likely LoF
2A-R504W	1.2	1.3	1.2	1.1	M (1.6)	H (0.36)	Conflict	0.91, 1.3	Indeterminant
2A-V506A	M (1.7)	1.1	M (2.1)	1.1	1.4	1.1	0,2	4.8, 9.1	Possible GoF
2A-S545L	0.69	0.79	0.81	M (0.53)	0.94	0.89	Subthreshold	0.51, 0.34	Possible LoF
2A-E551K	H (6.7)	H (4.6)	0.86	H (2.0)	H (3.0)	0.68	4,0	4.9, 19	Likely GoF
2A-P552R	H (11)	H (23)	M (0.65)	1.2	H (15)	1.3	Conflict	15, 26	Possible GoF
2A-S554T	H (4.6)	H (5.2)	M (1.6)	H (4.1)	H (3.2)	1.4	4,1	14, 43	Likely GoF
2A-L611Q	M (2.0)	1.3	H (> 43)	1.2	H (2.3)	0.73	2,1	55, 41	Likely GoF
2A-N615K	M (1.8)	M (1.5)	H (> 43)	M (0.67)	1.2	0.74	Conflict	24, 41	Possible GoF
2A-K669N	H (3.1)	H (3.9)	1.0	H (0.22)	H (4.3)	0.97	Conflict	0.63, 1.7	Indeterminant
2A-P699S	M (1.5)	0.92	1.2	0.89	0.93	0.83	Subthreshold	0.70, 2.3	No Effect
2A-M705V	M (0.60)	1.2	M (1.9)	H (0.41)	1.0	0.68	Conflict	0.35, 0.38	Possible LoF
2A-A716T	H (0.17)	0.92	1.4	0.89	M (0.57)	H (0.40)	2,1	0.21, 0.76	Likely LoF
2A-D731N	H (0.001)	0.80	0.81	H (0.21)	H (0.25)	H (0.22)	4,0	0.0003, 1x10[7]	Likely LoF
2A-V734L	M (0.67)	0.92	0.87	0.90	M (0.54)	0.95	0,2	0.52, 0.23	Possible LoF
2B-E413G	H (0.019)	1.2	1.1	1.0	H (0.04)	H (0.18)	3,0	0.008, 0.001	Likely LoF
2B-C461F	H (0.009)	H (2.5)	1.0	1.0	H (0.05)	H (0.16)	Conflict	0.009, 0.0004	Possible LoF
2B-S541G	H (4.3)	H (4.8)	1.1	M (1.8)	H (2.3)	M (0.60)	Conflict	1.8, 4.4	Possible GoF
2B-S541R	H (0.14)	H (0.14)	M (0.67)	M (0.54)	H (0.13)	1.1	3,2	0.03, 0.004	Likely LoF
2B-R540H	M (2.3)	M (1.5)	0.89	H (2.0)	H (2.0)	H (0.43)	Conflict	1.0, 1.6	Indeterminant
2B-P553T	M (0.52)	H (0.31)	0.93	1.0	H (0.19)	1.2	2,1	0.11, 0.29	Likely LoF
2B-S555N	H (3.6)	H (2.8)	M (1.5)	M (1.8)	H (2.3)	0.97	3,2	4.9, 15	Likely GoF
2B-W607C	0.75	0.98	H (> 42)	H (0.32)	M (0.61)	0.84	Conflict	7.0, 5.9	Possible GoF
2B-G611V	0.88	M (1.5)	H (> 42)	M (0.54)	M (1.5)	1.2	Conflict	28, 12	Possible GoF
2B-N615I	1.2	M (2.0)	H (> 42)	M (0.54)	0.88	0.79	Conflict	17, 27	Possible GoF
2B-N615K	1.1	M (1.7)	H (> 42)	M (0.54)	1.2	M (0.53)	Conflict	21, 27	Possible GoF
2B-N616K	1.4	H (3.1)	H (> 42)	M (0.54)	1.3	1.1	Conflict	50, 37	Possible GoF
2B-V620M	1.4	0.74	H (6.4)	M (1.5)	M (1.6)	M (0.58)	Conflict	8.6, 9.9	Possible GoF
2B-R696H	H (4.5)	0.86	0.93	1.0	H (3.6)	0.75	2,0	1.5, 4.8	Likely GoF
2D-S573F	1.3	1.3	M (0.58)	H (0.24)	0.73	M (0.60)	1,2	0.04, 0.11	Likely LoF
2D-L670F	H (3.3)	1.3	1.3	H (54)	H (6.7)	M (0.62)	Conflict	148, 69	Possible GoF
2D-S1271L	M (1.6)	M (0.50)	1.4	H (0.48)	0.91	M (0.69)	Conflict	0.37, 0.73	Possible LoF
2D-R1313W	1.0	M (0.52)	1.2	H (0.37)	0.89	H (0.33)	2,1	0.12, 0.18	Likely LoF

GluN1 variants were co-expressed with GluN2A. Glutamate and glycine potency ratios are given as WT/variant EC_{50} because EC_{50} is reciprocally related to potency. Mg^{2+} IC_{50}, open probability, weighted tau (τ_W), and surface expression fold effects are given as variant/WT. A high (H) or moderate (M) confidence (see Table 5) for the change is indicated; red is LoF and blue is GoF. The number of high (H) and moderate (M) changes are given when there are no conflicts. Conflicting and subthreshold variants were promoted to *Possible GoF* or *Possible LoF* if the fold change in the synaptic (left number) or non-synaptic (right number) charge transfer change was >2.5-fold or <0.4-fold (Supplemental Table S2). For some variants (Supplemental Table S4), we added new experiments for one parameter.

Supplemental Table S4. New data recorded to supplement published data

Variants	Mg^{2+} IC_{50}, µM [95% CI], (n)	Current Response (1 mM Mg^{2+}) / Current Response (0 mM Mg^{2+})
WTGluN1/WT 2A	30 [30, 31], (51)	3.1 ± 0.06% (50)
2A-G483R	29 [20, 42], (5)	1.2 ± 0.69% (5)
2A-R504W	33 [23, 47], (6)	4.2 ± 0.59% (6)
2A-V506A	57 [33, 98], (5)	8.0 ± 2.1% (5)
2A-K669N	19 [15, 49], (4)	2.5 ± 1.1% (3)
2A-P699S	34 [27, 44], (7)	3.1 ± 0.50% (7)
2A-M705V	57 [43, 76], (6)	3.9 [a]
2A-A716T	37 [27, 49], (11)	2.9 ± 0.80% (11)
2A-V734L	18 [11, 28], (15)	3.5 ± 0.80% (15)
WTGluN1/WT 2B	27 [26, 28], (26)	4.2 ± 0.13% (26)
2B-E413G	26 [21, 32], (17)	5.8 ± 0.40% (9)
2B-C461F	28 [18, 45], (12)	5.2 ± 1.7% (12)
2B-R540H	21 [16, 28], (10)	2.4 ± 0.83% (9)
2B-R696H	22 [15, 30], (9)	2.4 ± 1.1% (8)

The fitted IC_{50} value for Mg^{2+} determined using two electrode voltage clamp recordings from oocytes (V_{HOLD} -60 mV) is given for same day WT controls and variant NMDA receptors. The 95% confidence interval is given in square brackets [95% CI] and the number of oocytes given in parentheses (n). The ratio of the current response at -60 mV in the presence and absence of 1 mM Mg^{2+} is given as mean ± SEM% (n). [a] Mg^{2+} inhibition at 1 mM calculated from the fitted curves.

Supplemental Table S5. Summary of *GRIN* variants with no detectable current response in both *Xenopus* oocytes and transfected HEK cells

Variants	Glutamate EC_{50}	Glycine EC_{50}	Mg^{2+} IC_{50}	P_{OPEN}	Tau	Surface expression	Net Call	PMID	Resource
**2A-R518H**	ND	ND	ND	ND	ND	0.50	Likely LoF*	27839871	Swanger 2016
**2A-T531M**	ND	ND	ND	ND	ND	0.17	Likely LoF*	27839871	Swanger 2016
**2A-A548P**	ND	ND	ND	ND	ND	1.0	Likely LoF*	37000222	Xie 2023
**2B-C436R**	ND	ND	ND	ND	ND	0.12	Likely LoF*	27839871	Swanger 2016
**2B-A549V**	ND	ND	ND	ND	ND	1.0	Likely LoF*	37000222	Xie 2023
**2B-F550S**	ND	ND	ND	ND	ND	0.90	Likely LoF*	37000222	Xie 2023
**2B-L551S**	ND	ND	ND	ND	ND	0.91	Likely LoF*	37000222	Xie 2023
**2B-S555I**	ND	ND	ND	ND	ND	1.0	Likely LoF*	37000222	Xie 2023
**2B-P553L**	ND	ND	ND	ND	ND	0.67	Likely LoF*	28095420	Ogden 2017
**2B-A636P**	ND	ND	ND	ND	ND	0.068	Likely LoF*	28377535	Platzer 2017

ND: No detectable current, that is, the current amplitude was too small to measure.

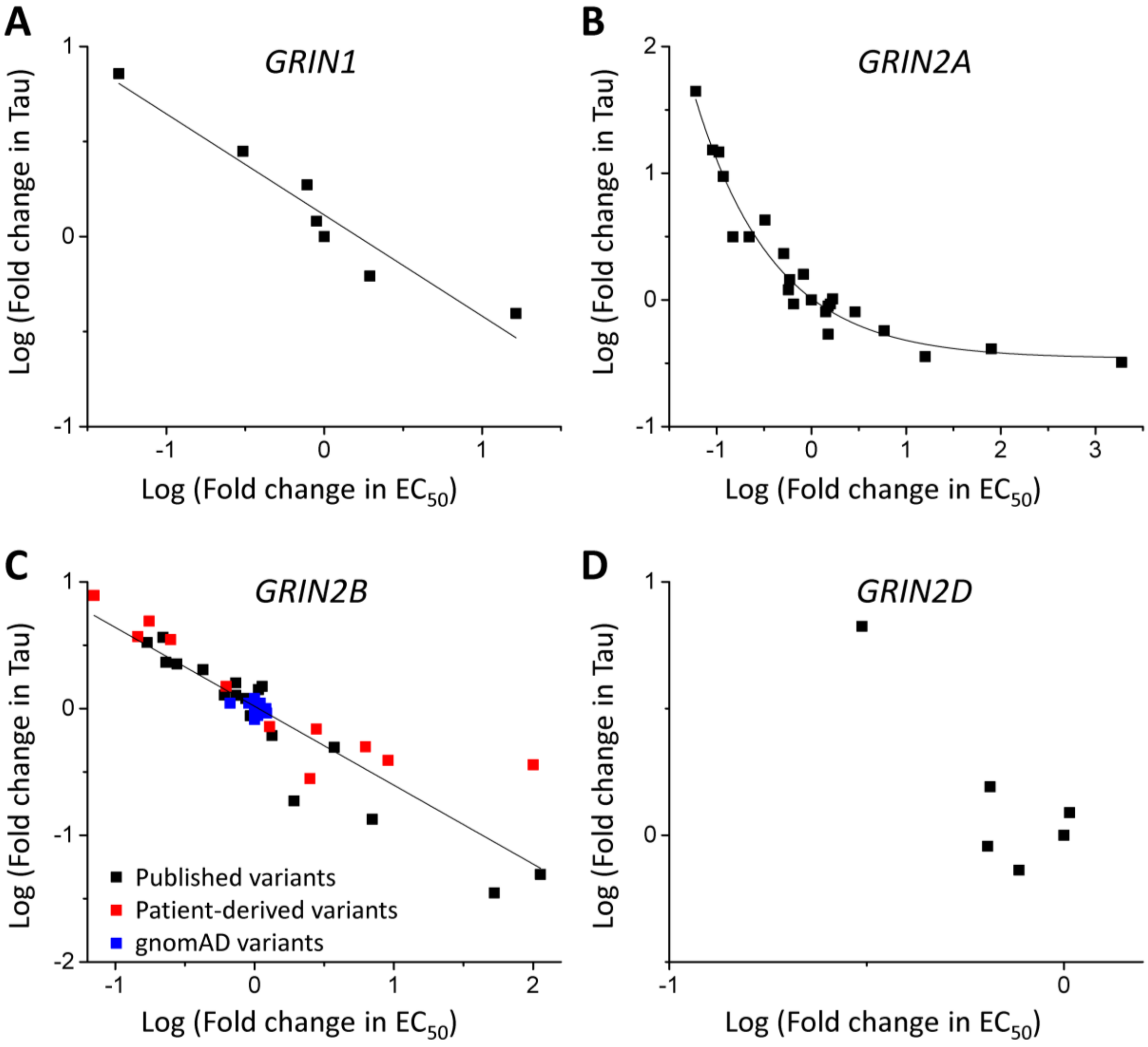

Supplemental Figure S1: The relationship between log(fold shift in glutamate EC_{50} calculated as variant/WT) and log (fold shift in weighted tau deactivation calculated as variant/WT). The log(fold shift in glutamate EC_{50}) was determined in oocytes and log (fold shift in tau deactivation) was determined in HEK cells. The relationship can be empirically approximated either by a linear (*GRIN1*, *GRIN2B*) or an exponential function (*GRIN2A*), which allows estimation of fold changes in tau deactivation from fold changes in EC_{50} for (**A**) *GRIN1* variants (co-expressed with WT *GRIN2A*/GluN2A), (**B**) *GRIN2A* variants, and (**C**) *GRIN2B* variants. For *GRIN2B*, GluN2B-V821F was omitted because the small current amplitude complicated determination of tau and GluN2B-G689S was omitted because the large reduction in potency made determination of EC_{50} ambiguous. Black symbols are published variants, red symbols are patient-derived variants, and blue symbols are variants in gnomAD. There is not enough data for *GRIN2D* variants (**D**) to determine the relationship. These relationships are an approximation, and not as reliable as direct measure of tau deactivation, given that other factors can influence the time course for deactivation. The fold change in weighted tau deactivation for *GRIN1* and *GRIN2B* variants can be empirically estimated by

Equation S1: Fold change in tau = $10^{(\textit{slope} \times \log(\textit{fold change in EC50}) + \textit{intercept})}$

where *slope* and *intercept* were determined by linear regression. For *GRIN1*, the slope was -0.5319 and intercept was 0.1137 (R^2=0.91). For *GRIN2B,* the slope was -0.6226 and intercept was 0.01787 (R^2=0.81). The fold change in weighted tau deactivation for *GRIN2A* variants can be empirically estimated by

Equation S2: Fold change in tau = $10^{(-0.4629 + 0.4749 \exp(-\log(\textit{fold change in EC50}) / 0.8370))}$

References

Li J, Zhang J, Tang W, Mizu RK, Kusumoto H, XiangWei W, Xu Y, Chen W, Amin JB, Hu C, Kannan V, Keller SR, Wilcox WR, Lemke JR, Myers SJ, Swanger SA, Wollmuth LP, Petrovski S, Traynelis SF, Yuan H (2019) De novo GRIN variants in NMDA receptor M2 channel pore-forming loop are associated with neurological diseases. *Human Mutation* 40 (12): 2393-2413. doi:10.1002/humu.23895

Chen W, Tankovic A, Burger PB, Kusumoto H, Traynelis SF, Yuan H (2017) Functional Evaluation of a De Novo GRIN2A Mutation Identified in a Patient with Profound Global Developmental Delay and Refractory Epilepsy. *Mol Pharmacol* 91: 317-330.

Ogden KK, Chen W, Swanger SA, McDaniel MJ, Fan LZ, Hu C, Tankovic A, Kusumoto H, Kosobucki GJ, Schulien AJ et al. (2017) Molecular Mechanism of Disease-Associated Mutations in the Pre-M1 Helix of NMDA Receptors and Potential Rescue Pharmacology. *PLoS Genet*, 13: e1006536

Swanger SA, Chen W, Wells G, Burger PB, Tankovic A, Bhattacharya S, Strong KL, Hu C, Kusumoto H, Zhang J, Adams DR, Millichap JJ, Petrovski S, Traynelis SF, Yuan H (2016) Mechanistic insight into NMDA receptor dysregulation by rare variants in the GluN2A and GluN2B agonist binding domains. *American Journal of Human Genetics* 99(6): 1261-1280. doi:10.1016/j.ajhg.2016.10.002

Traynelis SF, Burgess MF, Zheng F, Lyuboslavsky P, Powers JL. (1998) Control of voltage-independent zinc inhibition of NMDA receptors by the NR1 subunit. *J Neurosci*. 18(16): 6163-75. doi: 10.1523/JNEUROSCI.18-16-06163.1998

Xie L, McDaniel MJ, Perszyk RE, Kim S, Cappuccio G, Shapiro KA, Muñoz-Cabello B, Sanchez-Lara PA, Grand K, Zhang J, Nocilla KA, Sheikh R, Armengol Dulcet L, Romano R, Pierson TM, Yuan J, Myers SJ, Traynelis SF (2023) Functional effects of disease-associated variants reveal that the S1-M1 linker of the NMDA receptor critically controls channel opening. *Cellular and Molecular Life Sciences* 80(4): 110. doi: 10.1007/s00018-023-04705-y

Xu Y, Song R, Chen W, Strong K, Shrey D, Gedela S, Traynelis SF, Zhang G, Yuan H (2021) Recurrent seizure-related GRIN1 variant: Molecular mechanism and targeted therapy. *Ann Clin Transl Neurol* 8(7): 1480-1494. doi: 10.1002/acn3.51406

Yuan H, Hansen KB, Zhang J, Pierson TM, Markello TC, Fajardo KV, Holloman CM, Golas G, Adams DR, Boerkoel CF et al. (2014) Functional analysis of a de novo GRIN2A missense mutation associated with early-onset epileptic encephalopathy. *Nature Communications* 5: 3251.
